# Self-Healing Hydrogels: Preparation, Mechanism and Advancement in Biomedical Applications

**DOI:** 10.3390/polym13213782

**Published:** 2021-10-31

**Authors:** Anupama Devi V. K., Rohin Shyam, Arunkumar Palaniappan, Amit Kumar Jaiswal, Tae-Hwan Oh, Arputharaj Joseph Nathanael

**Affiliations:** 1 Tissue Engineering Group, Centre for Biomaterials, Cellular and Molecular Theranostics (CBCMT), Vellore Institute of Technology (VIT), Vellore 632014, Tamil Nadu, India; anupamadevi.vk@vit.ac.in (A.D.V.K.); rohin.shyam@vit.ac.in (R.S.); arunkumar.p@vit.ac.in (A.P.); 2 School of Bio Sciences and Technology (SBST), Vellore Institute of Technology (VIT), Vellore 632014, Tamil Nadu, India; 3 School of Chemical Engineering, Yeungnam University, Gyeongsan 38541, Korea; taehwanoh@ynu.ac.kr

**Keywords:** self-healing, dynamic bonds, hydrogels, wound healing, drug delivery, cell delivery, tissue engineering

## Abstract

Polymeric hydrogels are widely explored materials for biomedical applications. However, they have inherent limitations like poor resistance to stimuli and low mechanical strength. This drawback of hydrogels gave rise to ‘‘smart self-healing hydrogels’’ which autonomously repair themselves when ruptured or traumatized. It is superior in terms of durability and stability due to its capacity to reform its shape, injectability, and stretchability thereby regaining back the original mechanical property. This review focuses on various self-healing mechanisms (covalent and non-covalent interactions) of these hydrogels, methods used to evaluate their self-healing properties, and their applications in wound healing, drug delivery, cell encapsulation, and tissue engineering systems. Furthermore, composite materials are used to enhance the hydrogel’s mechanical properties. Hence, findings of research with various composite materials are briefly discussed in order to emphasize the healing capacity of such hydrogels. Additionally, various methods to evaluate the self-healing properties of hydrogels and their recent advancements towards 3D bioprinting are also reviewed. The review is concluded by proposing several pertinent challenges encountered at present as well as some prominent future perspectives.

## 1. Introduction

Hydrogels are insoluble, chemically or physically crosslinked, three-dimensional (3D) networks that swell and retain significantly in an aqueous solution. This exceptional ability to absorb water into the entwined structure makes it a source of considerable interest in scientific research, especially biomedical applications [1]. Various properties of the hydrogel like biocompatibility, biodegradability, elasticity, hydrophilicity, and its adhesive property have caused it to excel in the biomedical field, but its poor mechanical properties have hampered its advancement [2]. Since the irreversible covalent cross-links prevent the material from healing after a breach, such hydrogels are unsuccessful at sustaining their function or maintaining their desirable mechanical properties [3].

**Figure 1:**
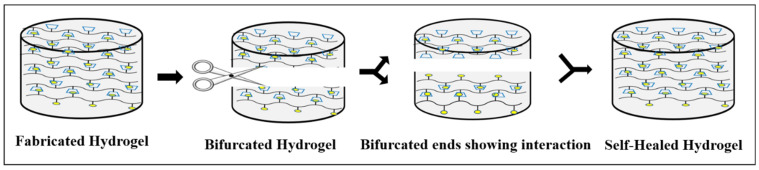
Demonstration of the healing process in a hydrogel.

After mechanical damage, self-healing hydrogels spontaneously regenerate through dynamic covalent cross-linkages, covalent, and non-covalent interactions. Thus, the idea of self-healing hydrogels aroused interest, inspired by natural organisms where the function of self-healed material could be maintained by inducing it through external stimuli or interactions that have been discussed in detail so far [4]. As these self-healing hydrogels has the ability to repair itself, it exhibits a prolonged life span even after being exposed to external forces [5]. As a result, self-healing hydrogel is always accompanied by injectability, which reduces comfort and pain while maintaining the wound in its natural state and extending the life span of the dressing material [6]. These self-healing hydrogels called “smart hydrogels” have the capability of automatically repairing themselves either completely or partially after damage (Figure 1). This feature has piqued interest towards its application in biomedical research because it has the potential to extend the longevity of biomaterials while also retaining their original properties, thereby being cost effective [7]. Furthermore, it can improve the material’s reliability and safety in specific applications by preventing failures caused due to wear and tear [8]. This results in its wide variety of applications in biomedical sectors, including drug delivery systems, tissue engineering, and wound healing [9].

However, complicated preparation techniques or the use of potentially toxic crosslinking chemicals may limit the production of self-healing hydrogels on a larger scale. Meanwhile, these hydrogels are mostly sought after in the biomedical field. Despite there being few reviews of self-healing hydrogels in the literature [4,10,11,12,13], this review is unique in several aspects. This review first outlines the different self-healing mechanisms like both physical and chemical crosslinking methods and the impact of nanotechnology in self-healing hydrogel fabrication. This is followed by various techniques to evaluate the self-healing properties of the hydrogel. The second section of the paper delves into the significance of these self-healing hydrogels in the production of wound dressing material for accelerating wound healing process and providing a barrier against bacterial colonization in wound environment. In addition, a comprehensive evaluation of the use of self-healing hydrogels for controlled drug delivery, as well as combination therapy involving dual drug delivery systems or drug with additional agents such as magnetic or photothermal agents, has been discussed. Furthermore, the impact of self-healing hydrogels on cell development and differentiation is covered in depth. In addition, the impacts of self-healing hydrogels on bone and cartilage tissue engineering are explored towards the end of the paper. Finally, the unresolved problems in the manufacturing of such hydrogels will be explored, as well as their current limitations and future potential in biomedical research.

## 2. Self-Healing Mechanism

**Figure 2:**
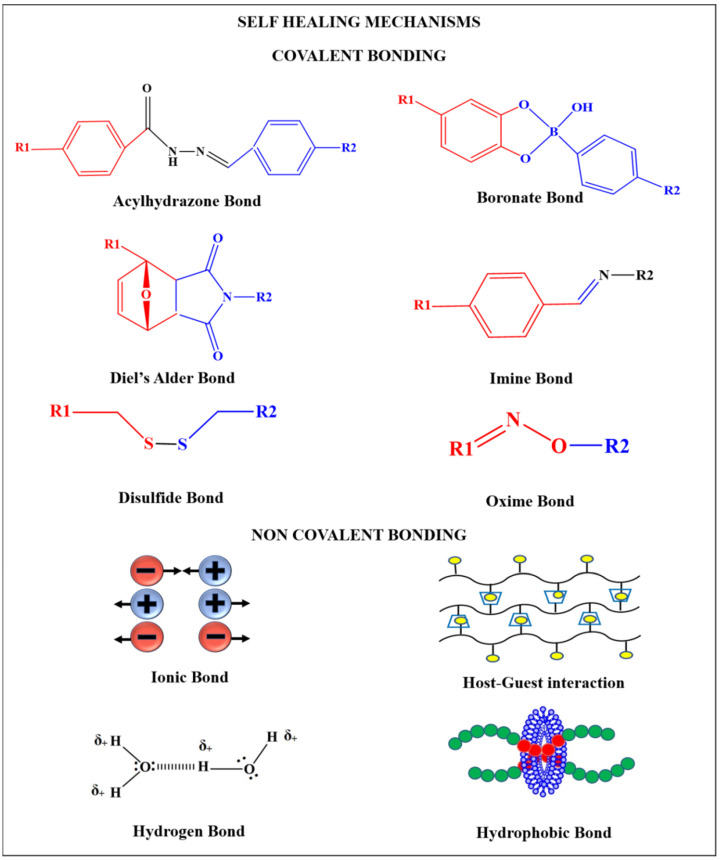
Bonds involved in self-healing mechanisms.

Various mechanisms have been followed to obtain self-healing hydrogels. The healing mechanism is broadly classified as—covalent and non-covalent bonding (Figure 2). Dynamic covalent bonding includes imine bonds, boronate bonds, Diels-Alder reaction, acylhydrazone bonds, oxime bonds, and disulfide bonds whereas non-covalent interaction includes hydrogen bonds, ionic interaction, host guest interaction, and hydrophobic interaction. The hydrogels obtained from non-covalent interactions are generally highly flexible and self-heal because of their ability to easily break and reconstruct crosslinks, whereas those obtained from covalent bonding are highly stable [2,3].

### 2.1. Hydrogen Bonding

The crosslinked hydrogels based on hydrogen bonds are obtained through reversible cross-linking of polymeric networks where hydrogen atoms interact with highly electronegative atoms such as nitrogen, oxygen, and fluorine. These hydrogels exhibit improved bond strength and self-healing ability. The self-healed hydrogels fabricated via the hydrogen bonding mechanism are relatively-less stable, compared to self-healing hydrogel prepared utilizing ionic and covalent interactions. Ye et al. has shown that the incorporation of hydrophobic water-shielding groups maintained hydrogel’s stability, resulting in a self-healing hydrogel comprising of cytosine, guanosine, and modified hyaluronic acid that did not require the use of healing agents [14]. The conductive hydrogel was synthesized from guar gum, a water-soluble galactomannan, combined with acidic poly(3,4-ethylenedioxythiophene): poly(styrenesulfonate) to promote wound closure and tissue rearrangement to speedup wound healing [6]. The freeze-thawing approach has been used to create self-healing polyvinyl alcohol (PVA) hydrogel that forms hydrogen bonding at the fractured site due to the presence of sufficient free hydroxyl groups in the PVA chain [7].

Polyvinyl alcohol, 3,4-dihydroxyphenyl-*L*-alanine (DOPA) and iron complex showed rapid healing efficiency after disintegration and it has been evaluated by continuous strain sweep test. DOPA’s catechol groups play an important part in self-healing activities [15]. At room temperature, supramolecular hydrogels including poly(ethylene oxide), poly(*N*-isopropylacrylamide) (PNIPAm), and ureido pyrimidinone (UPy) resulted in a higher self-healing ability. This has been achieved due to the presence of PNIPAm segments in the hydrogel, which gets dehydrated and tends to form micellar shapes during breakage. This creates a hydrophobic environment that enhances the UPy dimerization to promote reversible supramolecular assembly by hydrogen bond cross-linking [16]. The poly *N*-acryloylglycinamide building blocks for supramolecular self-healing hydrogels with dual amide motifs were shown to have good mechanical strength and a maximum healing efficacy of 84%. The maximum tensile strength of self-healed hydrogel has been reported to be 1.1 MPa, with a Young’s modulus of 150 kPa [17].

### 2.2. Ionic Bonds

The interaction between oppositely charged polymers promotes crosslinking in polymeric solutions during the synthesis of hydrogels. The migration of free ions to the non-crosslinked or broken region of the polymeric chain causes self-healing, resulting in a reversible reaction caused by ionic bonding between free ions and polymers. For example, using Fe ions in the production of hydrogels to generate self-healing hydrogels based on ionic bonding. For example, an ionic bond achieved by interaction of carboxylic group present in polyacrylic acid (PAA) with Fe^3+^ ions lead to the self-healing ability of the hydrogel. This hydrogel exhibited self-healing ability because of the existence of ionic interaction and an improved mechanical strength due to the formation of covalent bonds by the carboxylic acid ions present in PAA [18]. PAA-Fe^3+^ hydrogel thus obtained underwent healing for 24 h at room temperature and on cyclic stretching relaxation test, it exhibited good stability that could withstand up to 200% stretch of its original length even after 1000 cycles [19]. Fe^3+^ also acted as a crosslinker in a nanocomposite hydrogel that has self-healing ability and enhanced mechanical strength. It revealed tensile strength of 860 kPa for prepared hydrogel and 560 kPa for self-healed hydrogel. The properties such as self-repair, toughness, stretchability makes these hydrogels unique for tissue engineering applications [18]. Likewise, catechol has also been used for making ionic bond-based hydrogels. These self-healing and load-bearing hydrogels were created by electrostatic crosslinking Fe^3+^ ions and catechol-grafted chitosan, which re-established their full strength within 100 s in cyclic time sweep experiments [20]. Similarly, dopamine and catechol groups grafted on the surface of montmorillonite hydrogel were prepared, which showed 70% recovery of its original storage moduli even after applying 100% amplitude oscillatory force. Ionic interaction of catechol and ferric ions is responsible for the self-healing property [21]. In addition, a carboxybetaineacrylamide self-healing hydrogel containing zwitterionic material that was repaired via ionic bonds recovered 90% of its compressive capabilities following repair and showed time-independent healing behavior [22].

### 2.3. Host-Guest Interactions

Selective complementary interactions of supramolecular materials with host-guest relations make this interaction more unique in biomaterials application. Various guest groups currently in use are adamantane, ferrocene, azobenzene, cholic acid, and cholesterol exhibiting a promising future in biomedical research [23]. Masaki et al. developed redox responsive PAA hydrogels, with 6-cyclodextrin (6CD) acting as a host molecule and ferrocene (Fc) functionalized in the host material. The ferrocene is responsible for the redox responsive properties of the prepared hydrogel. The self-healing property of the hydrogel was tested using the step strain rheology measurement test which showed that the hydrogel recovered a maximum of 90% of their initial G’ value even after the polymeric network of hydrogels distorted at 200% strain. This reconstruction could be achieved by the host-guest affinity between the βCD and Fc [24]. Hydrogels based on B-cyclodextrins exhibit good self-healing properties. Adamantine (Ad) is another type of molecule which has similar properties to Fc that forms an inclusion complex with βCD. Supramolecular hyaluronic acid grafted with βCD and Ad hydrogel at room temperature exhibited rapid self-healing ability and shear-thinning behavior [23]. Due to their ability to quickly restore the structure, these hydrogels could be employed as inks and support matrices in 3D printing [25]. To increase toughness and elastic moduli, methacrylation of hyaluronic acid resulted in hyaluronic acid-methacrylate (MeHA). CD-Ad was used to create a dual crosslinking hydrogel such as CD-MeHA and Ad-MeHA, that forms a host-guest complex and re-duces self-healing time to approximately 1 s [26]. Furthermore, layer-by-layer assembly of CD-MeHA/Ad-MeHA hydrogels demonstrated a shear-thinning capability, which might be employed as ink for fabricating a layer-by-layer hydrogel [27]. Kohei et al. described a dual host-guest self-healing and shape-memorable hydrogel system that combines the β-cyclodextrin—adamantine and β-cyclodextrin—ferrocene complexes into a supramolecular network to produce stimuli-responsive multifunctional hydrogels [28]. Apart from this, the cholic acid with βCD [29], pluronic F180 with βCD [30] and α-bromonaphthalene polymer with βCD [31] inclusion complexes revealed self-healing behavior within a minute. The cationic CD oligomer was crosslinked with epichlorohydrin and modified with allyl glycidyl ether and glycidyl trimethylammonium chloride to overcome its low mechanical strength and stability thus making it suitable for wound healing application [32]. A shear-thinning hydrogel based on a cyclodextrin modified alginate and a methacrylated gelatin was created with potential in tissue engineering application as a result of the host-guest interaction between the hydrophobic cyclodextrin and aromatic remains of gelatin [33].

### 2.4. Hydrophobic Bonds

Generally, hydrophobic crosslinking is a reversible non-covalent interaction that explains the relation between water and hydrophobic monomers. This property could be used for designing the physical self-healing hydrogels through the self-assembling mechanism of monomers in an aqueous medium. Micellar polymerization is an extensively used method to develop hydrophobic bonds based self-healing hydrogels. This reaction occurs with the assistance of four crucial components known as hydrophobic units, hydrophilic units, surfactants, and electrolytes. In this mechanism, a surfactant is used for solubilizing the hydrophobic compounds and electrolyte-aqueous solutions assist the copolymerization along with hydrophilic compounds. Co-polymerization of stearyl methacrylate (C18-hydrophobic monomers), dodecyl acrylate and a hydrophilic monomer- acrylamide in the presence of surfactant sodium dodecyl sulfate (SDS) and sodium chloride (electrolyte) yielded the self-healable hydrogels. These hydrogels exhibited 100% healing efficiency at room temperature within seconds and the healed hydrogels attained maximum elongation of about 3600% [34,35,36]. SDS is responsible for solubilizing the assembly of C18 monomers, that resulted in the formation of a reversible micellar structure which gives the hydrogels the ability to repair. The electrostatic interaction has been diminished with the help of NaCl, that solubilizes the micellar growth [37]. One of the most important aspects in generating a self-healing hydrogel is the choice of hydrophilic monomers, which has a significant impact on the hydrogel’s self-healing and mechanical behavior. The combination of PAA, C18, SDS, and NaCl produced hydrogels with self-healing property and increased mechanical strength. The self-healing hydrogels showed an elongation ratio of 1800–5000%. The healing was about 60% at room temperature and 80% at 80 °C [38,39]. The elongation ratio at break is increased up to 4200% when PAA is replaced with poly (*N*, *N*-dimethylacrylamide) in C18, SDS, and NaCl solution [40]. Another significant parameter is the selection of hydrophobic monomers, which will greatly influence the mechanical strength of the hydrogels [41]. For example, the hydrophobic monomer known as fatty alcohol polyoxyethylene acrylate was used for producing self-healing hydrogel with improved mechanical strength up to 318 kPa and elongation ratios of 1000–3000% [38,39,40]. Similar to the SDS-NaCl hydrogel system, the cetyltrimethylammonium bromide and SDS combination produce self-healing with better elastic behavioral hydrogels. After 60 min of the healing process, they exhibited a healing efficiency of 98% and attained a maximum of 1800–5000% elongation ratios at break [39]. The acryloyl-6-aminocaproic acid hydrogel was fabricated which possesses a balance of hydrophilic and hydrophobic interaction thus resulting to be a rapid autonomous synthetic hydrogel [42].

### 2.5. Imine Bonds

**Figure 3:**
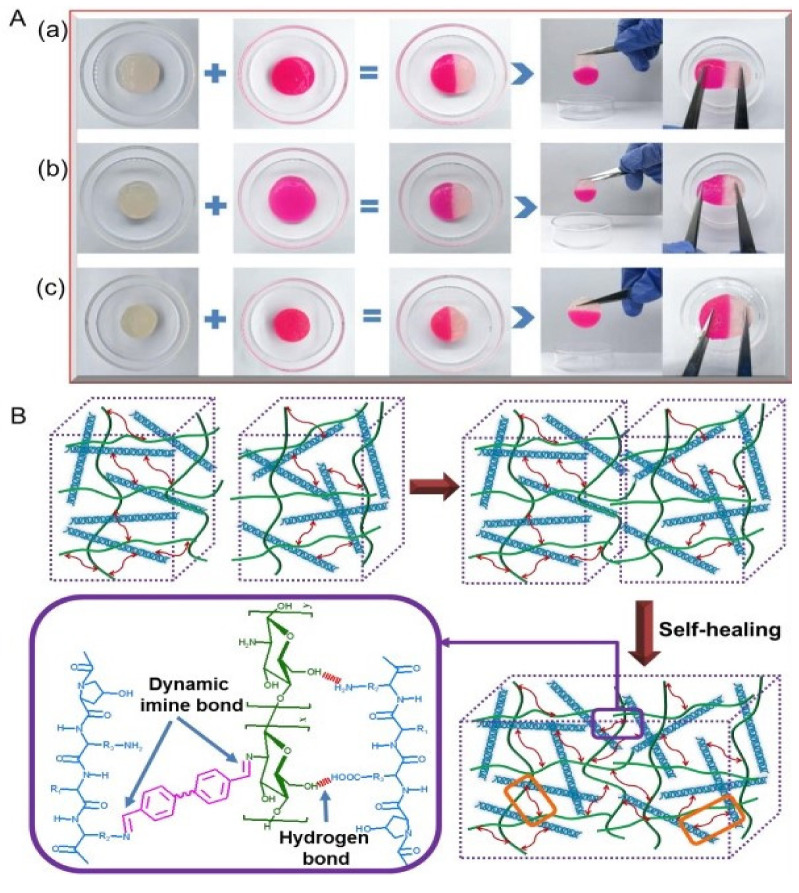
(**A**) Demonstration of Cut-Heal property of the hydrogel obtained from Collagen-chitosan at ratio of (**a**) 1/1, (**b**) 1/2 and (**c**) chitosan alone (**B**) Diagrammatic representation of Self-healing mechanism showing hydrogen bond and imine bond formation. (Reproduced with permission from Ref. [47]. Copyright 2020, American Chemical Society).

Imine bonds (i.e., Schiff base) are a promising class of dynamic covalent interaction that develops self-healing hydrogels by the formation of crosslinking among amine groups and aldehyde or ketone groups of polymers under physiological conditions [43,44]. The formation of Schiff base has greater reaction speed under mild conditions, ready gelation and heal ability, taking it forward for tissue engineering applications [45,46]. In addition, dibenzaldehyde-modified PFG_2000_, a modified PEG that acts as a telechelic crosslinker, has been used in the fabrication of a hybrid collagen chitosan hydrogel that revealed dynamic self-healing capabilities within 15 min of crack formation and entirely vanished within 45 min [47] (Figure 3).

Similarly, the hydrogel of agarose-ethylenediamine conjugate with dialdehyde-functionalized PEG (DF-PEG_2000_) was generated in 15 min owing to the quicker reaction of their respective amine and benzaldehyde groups, resulting in a potential hemostasis-maintaining hydrogel [48]. When two cut portions of injectable hydrogels were joined together under ambient settings, the injectable hydrogel made from aldehyde modified xanthan gum and phosphatidyl-ethanolamine, self-healed within 15 min [49]. Imine bond based self-healing hydrogels with unique material properties are produced by different polymers containing aldehyde groups interacting with chitosan. For example, injectable self-healing hydrogels were created by joining chondroitin sulfate multiple aldehydes and *N*-succinyl-chitosan [46] as well as *N*, *O*-carboxymethyl chitosan/oxidized chondroitin sulfate which showed antibacterial properties along with maintaining the hemostatic condition of the wound [50]. Similarly, the carboxymethyl chitosan incorporated with zinc, improved the antimicrobial effect of the hydrogel [51]. The oxidized form of green biological crosslinkers like konjac glucomannan along with chitosan followed Schiff- base interaction for the fabrication of hydrogel, resulted in high recovery of the hydrogel from breaking thereby revealing its self-healing ability [52]. Likewise, a natural wound dressing material was fabricated with chitosan and dialdehyde bacterial cellulose, showed good mechanical strength, injectability, drug delivery, antibacterial property, and a dynamic self-healing property due to the imine bond formation, thus making it a superior material for tissue engineering applications [45]. By combining glycol chitosan and telechelic difunctional poly (ethylene glycol) (DF-PEG), self-healing hydrogels were formed. The Schiff base linkage formed by the interaction between the NH_2_ groups of chitosan and the benzaldehyde groups of DF-PEG allows for the formation of self-healing hydrogels [53,54,55,56]. A hydrogel of quaternized chitosan (QCS) and benzaldehyde-terminated poly (ethyleneoxide)-b-poly (propyleneoxide)-b-poly (ethyleneoxide), pluronic^®^F127 (PF127) with outstanding mechanical properties, biocompatibility, and increased adhesiveness was also created [57]. The biocompatible complex hydrogel of aldehyde modified methyl cellulose and chitosan grafted polyethylene glycol loaded with exosomes showed an excellent effect in diabetic wounds [58]. The hydrogel formed by Schiff base reaction between -CHO group and -NH_2_ group of carboxymethyl cellulose dialdehyde and carboxymethyl chitosan with a dynamic strain of 250% [59] and also of *N*-carboxyethyl chitosan and PF127 showed a dynamic strain of 600% confirming the autonomous and efficient self-healing due to cross-linking [5]. *N*-carboxyethyl chitosan, adipic acid dihydrazide with hyaluronic acid-aldehyde was used by Haotian Bai et al. in the preparation of hydrogel that could promote stem cell proliferation with an effect on growth factor secretion thereby accelerating diabetic foot ulcers [60]. Another study by Xin Zhao et al. demonstrated a simple method of mixing quaternized chitosan-g-polyaniline and benzaldehyde group functionalized poly(-ethylene glycol)-co-poly(glycerol sebacate) with a critical strain of 250% for cutaneous wound healing applications [61]. 4-formylbenzoic acid modified ethanol amine grafted PLGA and propylene oxide modified chitosan resulted in composite self-healing hydrogels by Schiff base formation. [62]. Furthermore, oxidized alginate was combined with the natural polymer chitin, in its modified form called acrylamide modified chitin that comprises groups such as amino, carboxyl, and hydroxyl groups to create a hydrogel with significant biodegradability and biocompatibility making it potential in tissue engineering [63]. In addition to all these, gelatin-based hydrogels were fabricated with oxidized dextran and it conferred excellent self-healing property due to dynamic imine bonds [64]. Gelatin-based hydrogel based on Schiff’s base reaction is rare due to the non-appropriate amino groups in it. Thus, the facile approach that follows fabrication of hydrogel with ethylene diamine to increase the amino group of gelatins has been studied. It followed crosslinking with dialdehyde carboxymethyl cellulose thereby improved self-healing ability [65].

### 2.6. Disulfide Bond

In the presence of nucleophilic thiolates, the thiol/disulfide exchange process forms disulfide bonds in a neutral or alkaline environment [12,66] and it occurs at basic pH [8]. The disulfide bonds undergo an exchange reaction where S-S neighboring bonds get disrupt and reform through ionic intermediates [67]. Chen et al. produced a composite hydrogel consisting of thiolated polyethylene glycol and silver nitrate that resists external mechanical resistance and has antibacterial activity in the event of diabetic wound repair (AgNO_3_). The presence of Ag+ enhanced the bactericidal function, while the disulfide bond of Ag-S enhanced the self-healing ability, making it a viable material for diabetic wounds [68].

### 2.7. Acylhydrazone Bonds

Arylhydrazone bonds are created when aldehyde or ketone groups react with hydrazine groups to form a dynamic covalent bond [12,69] at acidic pH [8]. These bonds could be formed as a result of hydrolysis or exchange reactions [70]. The aldehyde groups of sodium alginate dialdehyde reacts with hydrazide and disulfide functional groups of 3,3′-dithiobis(propionohydrazide) functionalized poly(ethylene glycol) by Schiff base reactions to form acylhydrazone bonds. This leads to self-healing hydrogel with nearly 100% self-repair after breakage. This approach has been proposed with the utmost aim for its application in tissue engineering [71]. The acylhydrazone bonds formed by the reaction between aldehyde group and hydrazone bonds of oxidized dextran and acid dihydrazide respectively and gold standard antimicrobial agent Chlorhexidine acetate with prolonged release of bFGF resulted in improved wound healing [72]. In a novel study, the macroporous gelatin/oxidized sodium alginate/adipic acid dihydrazide hydrogel was obtained as an injectable self-healing hydrogel with microporous structure by the formation of azylhydrazone bonds between oxidized sodium alginate and adipic acid dihydrazide [73]. Similarly, the self-healing hydrogel with acylhydrazone bonds was fabricated under the ambient condition from the reaction of azylhydrazines from polyethylene oxide and aldehyde group of tris(4-formylphenoxy) methyl ethane [67].

### 2.8. Diels-Alder Reaction

The reversible click reaction that occurs between a conjugated diene and a dinophile especially an alkene or alkyne is called the Diels-Alder reaction [10,12]. It occurs without any catalyst or coupling reagent making it a suitable for biomedical applications in shear-thinning and self-healing hydrogels [74]. This chemistry was analyzed in the reaction of furyl modified cellulose nanocrystals and maleimide-end-functionalized PEG that showed about 78% healing efficiency due to the high content of DA bonds [70].

### 2.9. Boronate Bonds

The reversible covalent bonds formed by the combination of diols and boronic acid forms boronate bonds [10]. The pH-responsive hydrogel was fabricated from the cPEG polymer with 1,3-benzene diboronic acid at alkaline conditions through boronic ester bonds formations [75]. The diol borate ester bond between the borate ions with the -OH groups of guar gum in the collagen-based hydrogel for wound dressing material was investigated systematically [76].

### 2.10. Oxime Bonds

One of the ideal ways for the synthesis of hydrogel which possesses high reaction efficiency and ambient reaction conditions is oxime bonds that are formed by the reaction among hydroxylamine with the aldehyde or ketone groups [12,77]. The protein-polymer conjugates and cell surfaces commonly use these oxime bond formations [74].

## 3. Testing of Self-Healing Property

### 3.1. Common Testings of Self Healing Hydrogels

The fabricated hydrogel was tested for its self-healing ability by injecting it into water. The hydrogel regains back to its original form confirming its self-healing property [49]. Two equal pieces of disc-shaped hydrogel samples were placed together along its cut end at ambient conditions. The pieces merge at some point in time due to the dynamic bond formation [49]. A trace amount of dye like rhodamine R to differentiate one piece from the other has been used and the resultant self-healing ability has been examined visually [66].

**Figure 4:**
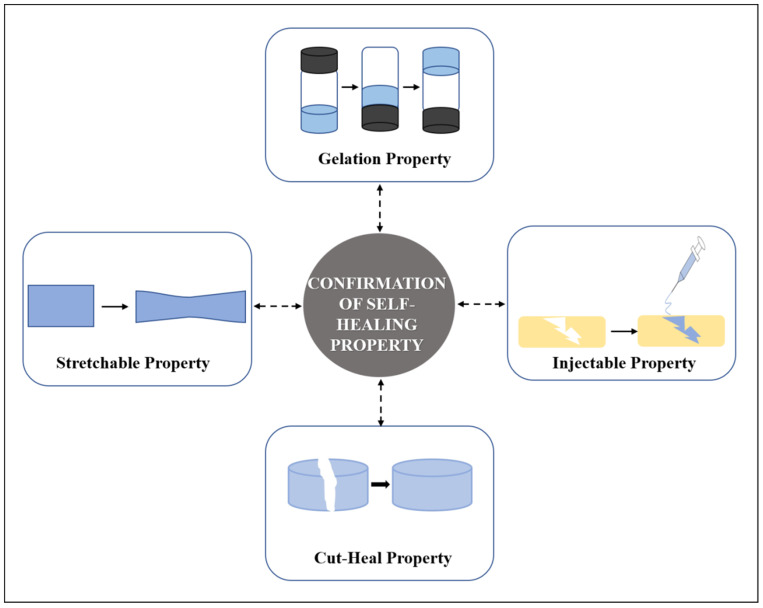
Confirmatory methods of self-healing property of a hydrogel.

Another simple test to demonstrate self-healing is the use of tweezers to stretch the hydrogel to check for the resistance against stretching and splitting thereby showing its strength to bear tensile force [64] (Figure 4). The scratch and heal method was followed by sprinkling a buffer solution with a pH of 7.4 and left to heal for 10 min. It was then visualized by an optical microscope [78].

### 3.2. Rheological Recovery Test

The internal recovery of materials is investigated by continuous step strain, where the larger strain is initiated for damage of materials while the storage modulus (G′) and loss modulus (G″) was recorded [79]. Strain amplitude test determines the critical point at which the hydrogel remains in the solid and liquid state thereby quantifies the self-healing ability [47]. The gelation process and mechanical property of hydrogel were investigated with a rheometer in a parallel configuration. Their dynamic time sweep experiment and dynamic oscillatory frequency sweep were also performed to examine its viscoelasticity [66,80].

## 4. Applications of Self-Healing Hydrogels

### 4.1. Self-Healing Hydrogels in Wound Healing

#### 4.1.1. Importance of Self-Healing Hydrogels in Wound Healing

In general, the surface wounds (i.e., epidermal injuries) are expected to heal by itself rapidly and the superficial wound (i.e., loss of epidermis and partial dermis layer) healing occurs through natural healing mechanisms. Initially, the mechanism occurs in a period with epithelialization in the edge of wounded skin by contracting the wounded tissue which sometimes promotes the functional defects. However, in case of deep wounds which are more than 1 mm diameter, the natural healing process is interrupted and cannot be epithelized on its own due to the destruction of a complete systemic host defense mechanism which makes the wounded area more prone to microbial infection. This insufficient activity promotes the development of non-healing wounds [81,82].

**Figure 5:**
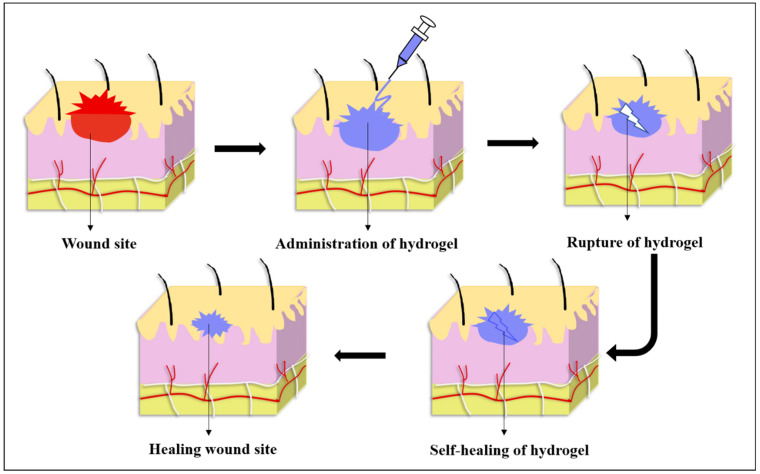
Application of self-healing hydrogel in wound healing.

Wound closure being the major step that promotes wound healing and prevents scar formation, sometimes cannot be achieved with the usage of staples and sutures that can cause detrimental effects to the surrounding tissues. This leads to the introduction of wound dressings such as hydrogels, films, etc., with improved functionalities [83]. The commonness of non-healing wounds in worldwide cases consistently rises as a result of lifestyles and population. In this scenario, the requirement of supporting wound dressing material known as synthetic skin grafts is looked to enhance the wound healing process. With the assistance of modern technological advancement, many wound dressing materials are commercially available in the wound care market in different forms namely hydrogels, scaffolds, injectable gels, and electrospun fibrous matrix [84,85,86]. The development of novel wound dressing materials is constantly increasing due to its rapid healing efficiency at a low-cost and affordable price with minimal discomfort and inconvenience to the patients (Figure 5).

Among other advanced dressing materials, hydrogels are considered to be a promising material in the wound care market owing to their peculiar material design and its excellent characteristics. Its characteristics include good biocompatibility, appropriate mechanical properties, flexibility, non-reactiveness with host tissues, the ability to absorb more wound exudates, and high water retention capacity. The property of water retention capacity helps to reduce the temperature and soothing the wound vicinity which prevents the formation of scabs [87,88,89]. As well, hydrogel allows fast wound healing by involving all four stages of the wound curative process known as hemostasis, inflammation, cell migration/proliferation and maturation [90]. Additionally, hydrogels promote wound healing by mimicking the natural tissues ECM like interconnected porous network. It is also an excellent carrier system for drugs/growth factors/proteins/cells [10,91,92]. Hydrogels possess high water content with a suitable swelling ratio, allows the exchange of gases, absorbs the exudates thereby promoting faster recovery. However, in the case of extremities like ankle, knee, wrists there is more possibility of discomfort and inconvenience on using the normal dressing materials.

Hence to overcome this, self-healing hydrogels acts as a superior choice as it withstands higher external resistance and heals on itself thus rendering stable connection between the bruises and the material [45,57]. Furthermore, hydrogels are easy to handle, the dressing can be easily changeable regularly without giving more pain to the patients. Currently, self-healing hydrogels got more attention in wound repair because of its intrinsic high structural stability after subjecting to the patients by maintaining the shape integrity even after mechanical destruction. Hydrogels having self-healing properties might be useful in wound healing and wound protection by preventing secondary injuries, extending the life of dressing materials, and providing additional protection for wound sites by retaining their shape through various self-repair mechanism [12,77,93].

Burn wounds are particularly complicated because they can cause damage to the tissue beneath the skin. In such cases, a standard wound dressing must be replaced after it absorbs the exudates, which can lead to complications such as wound exudates, wear and tear on the neo tissue formed in the wounded area [94]. In all such cases, the so-called “smart hydrogel” serves as a boon where the dissolvable capacity of the hydrogel overcomes the above-mentioned drawbacks of wear and tear making the procedure easy and painless.

#### 4.1.2. Advances of Self-Healing Hydrogels in Wound Healing

**Figure 6:**
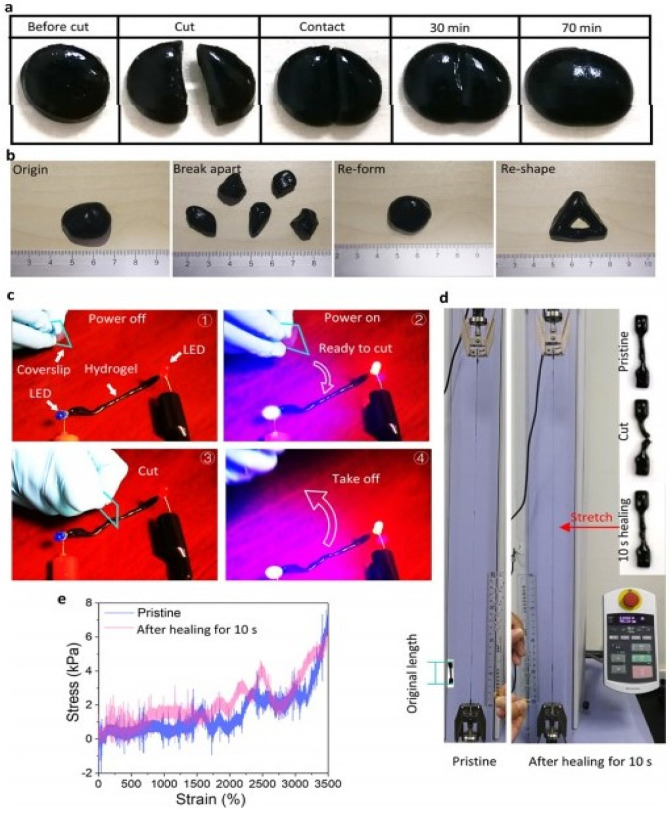
Methods to confirm self-healing ability of hydrogel (**a**) Cut heal ability within 70 min without stimuli (**b**) Shape changeable capability (**c**) Circuit obtained with the hydrogel that can selfheal (**d**) Stretchability performed with tensile testing apparatus with pristine and self-healed hydrogel (**e**) Stress-strain curves obtained from previous stretchability experiment. (Reproduced with permission from Ref. [99]. Copyright 2020, American Chemical Society).

New advances have always been initiated to improve the functionality and properties of wound dressing materials. A new initiative in self-healing hydrogel that promotes wound closure and healing has been demonstrated by Meng Li et al. with QCS, polydopamine-coated reduction graphene oxide and poly(*N*-isopropylacrylamide). It has the properties of adhesive property, conductivity and contractability that act as a multifunctional hydrogel with the combination of biomechanical and biochemical activity [83]. Diabetic wounds are always a concern because deficiency of growth factors and angiogenesis in diabetic patients results in the formation of chronic non-healing wounds. To tackle this, the research group of Qian, had demonstrated the self-healing hydrogel obtained as the combined effect of chitosan, silk fibroin and platelet-rich plasma which exhibited enhanced healing of wounds in a rat model that was induced with type 2 diabetics [95]. Tannic acid with poly (ethylene glycol) has now been proposed to be an injectable, self-healable combination that could facilitate rapid adhesion and enhanced wound healing [96]. Acryloyl-6-aminocaproic acid along with AA-g-*N*-hydroxysuccinimide has been developed into a promising injectable self-healable adhesive for the wound that has enriched gelation time and biocompatibility [97]. Chronic wounds, such as those found in diabetic patients, take a long time to heal, hence exosomes and other vesicles are used as vehicles for drug delivery to speed up the healing process. Wang et al. developed a pH-responsive multifunctional hydrogel comprised of Pluronic F127, oxidative hyaluronic acid, and Poly—*L*-lysine with the added benefit of release from exosomes were designed for chronic wound application [98]. In recent research, Zhang et al. have synthesized a self-healing hydrogel from polypyrrole/Zn-functionalized chitosan molecules and poly (vinyl alcohol) that could promote healing of chronic wounds and also assist in determining the strain and temperature [99] (Figure 6). As a result, the hydrogel’s self-healing capability is still being tweaked across its chemical, mechanical, and biological properties, overcoming the market’s current wound dressing limits.

#### 4.1.3. Mussel Inspired Self-Healing Hydrogels for Wound Healing

**Figure 7:**
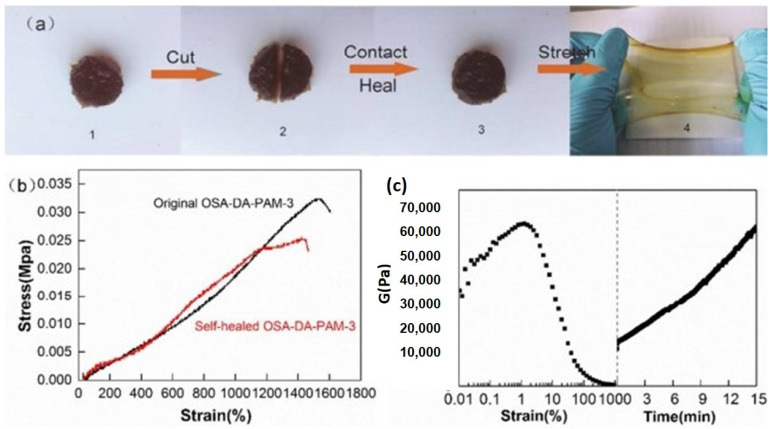
Methods for confirming self-healingability (**a**) 1, 2, 3—*c*ut-*h*eal ability, 4—*s*tretchability (**b**) *s*tress-strain curves of the fabricated hydrogel compared with the self-healed hydrogel (**c**) *r*heological property of hydrogel. *(*Reproduced with permission from Ref. [100]. Copyright 2018, American Chemical Society).

The mussel inspired dopamine has emerged as one of the most promising material due to its adhesive property in the field of biomedical research. Tao Chen et al. has synthesized a hydrogel out of dopamine grafted with oxidized sodium alginate and acrylamide that showed dynamic cross-linking through Schiff’s base reaction and recovered with 15 min on applying a strain of 1000% [100] (Figure 7). To obtain a hydrogel with self-adhesive and conductive properties, the combination of chitosan and graphene oxide coated with polydopamine was formed leading to its conductive pathway [101]. The bionic hydrogel for wound healing with oxidized hyaluronic acid and dopamine grafted ε-polylysine was formed by the mild Schiff base reactions with antibacterial properties [102]. The multi-responsive pH-responsive hydrogel was synthesized by dopamine functionalized polyallylamine [103]. Mussel inspired catechol groups lead to the development of various self-healable wound dressings like polyacrylate and polymethacrylate functionalized with silyl catechol groups that undergo hydrogen bonding in an acidic medium [75]. In another method, poly(acrylic acid)−poly-(acrylamide)−poly(dopamine) hydrogel has been synthesized with the adhesive property due to polyacrylamide microgel and non-covalent interactions which exhibited self-healing property and enhanced stretchability [104]. Mussel inspired self-healing hydrogels are thus playing a vital role in wound healing applications.

#### 4.1.4. Nanoparticles in Self-Healing Hydrogels for Wound Healing

The presence of metallic nanoparticles with other biomaterials helps in altering the original properties of the material. Zhang et al. had reported the fabrication of magnetic self-healing hydrogel by mixing carboxyl modified Fe_3_O_4_ nanoparticles with chitosan and DF-PEG material followed by Schiff base reaction showing recovery within the 60 s and providing 100% restoration [54]. Likewise, a hydrogel with copper nanoparticles embedded in the natural galactomannan guar gum was fabricated, which showed higher potential as a material for photothermal therapy and other biomedical applications [105]. Bioactive glass nanoparticles were also used to promote the healing ability as it promotes blood vessel formation. Thus, the bioactive glass containing copper was crosslinked with polyethylene glycol diacrylate and sodium alginate that exhibited efficient sealing of wounds, absorbed the wound exudates thereby acting as an excellent hydrogel for diabetic wound applications [106].

#### 4.1.5. Bacterial Cellulose Based Self-Healing Hydrogel for Wound Healing

Bacterial cellulose is an excellent hydrogel system used for wound healing, drug delivery, and tissue engineering applications [107,108,109]. Khamrai et al. developed a bio-derived self-healing wound healing patch made of curcumin loaded onto gelatin and ionically modified bacterial cellulose composite. The fabrication procedure was simple and environmentally friendly, and the addition of curcumin helped wound healing and offered antibacterial action [78]. Although many researchers have identified silver nanoparticles as a potential component for wound healing, the aforementioned group recently developed a strategy for ornamenting silver nanoparticles onto a modified form of bacterial cellulose, where the imine bonds in the covalently crosslinked patch aided in self-healing [110]. By enduring ionic interactions, the same researcher developed a polyelectrolyte film made of positively charged chitosan and negatively modified bacterial cellulose that exhibits dynamic self-healing properties. It was also established that the film has the potential to carry drugs and has wound-healing properties [111]. In addition, ionic bonding occurred as a result of the mechanism of polymerization triggered by UV irradiation onto cellulose grafted with polyacrylic acid and polyvinyl alcohol, leading to the formulation of the dynamic double networked antibacterial hydrogel. The pH responsiveness as well as its increased water retaining capacity has been discovered [112].

The modified form of cellulose known as carboxymethyl cellulose has been intensively researched, and it has demonstrated extraordinary stretching properties of around 2.5 times its original size, demonstrating its potential in wound healing applications [113]. Recently, a wound-dressing composite hydrogel constructed of polyvinyl alcohol-borax gel and reinforced with a composite combination of dopamine grafted with oxidized carboxymethyl cellulose and cellulose nanofibers demonstrated good antibacterial activity and self-healing capabilities. As a result of various covalent processes, it also demonstrated excellent mechanical properties [114]. It is also been made into an injectable nanofiber for wound healing, where it goes through a Schiff base reaction with sodium alginate that’s been functionalized with an aldehyde. It had excellent gelling and mechanical properties. The nanofiber was further examined in a rat model, demonstrating its promise in wound healing by assuring wound closure in a full thickness wound model [115].

#### 4.1.6. Leap towards 3D/4D Bioprinted Self-Healing Hydrogels

A bioink made of modified hyaluronic acid with adamantane or β-cyclodextrin that follows supramolecular assembly of host-guest complexes has been used in 3D bioprinting, where hyaluronic acid act as a backbone for the construct [25,27]. Furthemore, host-guest interaction of hyaluronic acid based multilayered constructs was formed with cucurbit[n]urils and 1,6- diamino hexane through 3D bioprinting [23]. The addition of methacrylate enables cross-link formation thus improving the mechanical properties of the hydrogel [79]. Amit Kumar Sharma et al. led a team of researchers who developed a hydrogel by 1:1:10 ratio of carboxymethyl cellulose, dialdehyde dextrin, and gelatin. The crosslinker was glutaraldehyde, and the binding agent was borax. It has high shear thinning and injectability qualities, allowing 3D printing a viable option for fabrication [116]. The 3D bio-ink was made with polyethylene glycol diacrylate, *N*-isopropyl acrylamide, *N*, *N*-methylene bis (acrylamide), 2-hydroxy-4-(2-hydroxyethoxy)-2-methylpropiophenone, and sodium alginate. The 3D printability of bioink was investigated, and it was also observed that it had an optimal swelling ratio, drug diffusion capability, and shape memory behavior, making it one of the promising uses in wound healing. In terms of self-healing and cell adhesion capabilities, more research is required to improve their role as 3D printed self-healing dressing material [117]. The 4D printing technology, which has a time dependence for fabricating structures, is one of the advanced 3D printing technologies. Several polymeric materials have been printed for tissue engineering applications. One of these is polyethylene glycol diacrylate, which increased cell adhesion and proliferation, laying the groundwork for future wound healing research [118]. Polycaprolactone, a self-healing polymer, has been intensively explored as a scaffold in 3D bioprinting for diverse uses. A group of researchers attempted to create a 4D printed scaffold with both self-healing and shape memory properties in an experiment. Polycaprolactone dimethacrylate with 2-ureido-4[1H]-pyrimidinone motifs has been utilized to achieve the scaffold. Further studies on cell adhesion and proliferation can be performed to explore it for application in wound healing [119].

### 4.2. Self-Healing Hydrogels in Drug Delivery

Drug delivery using conventional hydrogels (having static crosslinking bonds) are well known in the literature for many years [120,121]. In addition to their uncontrolled drug release kinetics, they also suffer from poor mechanical strengths due to the presence of high-water content [122,123,124]. In view of this, self-healing hydrogels are considered as promising candidates in the realm of delivering drugs or other bioactive agents in a controlled manner. These hydrogels can fix themselves from the fractures caused due to wear and tear and thereby could restore the original mechanical properties. This helps in preventing the leakage of drugs or other bioactive agents from the hydrogels, due to damages caused during their administration or other general wear and tear [124].

Self-healing hydrogels can reverse the damage caused to them through the reformation of cross-links among polymer chains within an appropriate time interval after breakage. The crosslinks could be non-covalent ones (involving reversible ionic bonds, hydrogen bonds, electrostatic interactions, hydrophobic or host-guest interactions, π-π interactions) or could be dynamic covalent bonds (involving imine groups, disulfide groups, boronate ester groups, acyl hydrazone groups, coordinate bonds and Diels-Alder reactions) as shown in Figure 2 [124].

#### 4.2.1. Self-Healing Hydrogels as Drug Delivery Platforms for Cancer Therapy

Cancer is one of the leading causes of mortality globally, accounting for about 10 million deaths in 2020 [125,126]. Despite advancements in treatment options and methodologies, solid tumors are difficult to treat clinically [127]. Surgical treatment for tumor debunking is the first line of treatment modality for the treatment of solid tumors [127]. In order to prevent local recurrence or metastasis of primary tumor, postsurgical treatment or adjuvant therapy is immediately followed up. One such treatment option is the local delivery of chemotherapeutic drugs in a controlled manner using various polymeric formulations [128]. Conventional hydrogels suffer from the problems of leakage of drugs, resulting in serious toxicity issues in the surrounding normal tissues. However, self-healing hydrogels have the positive aspects of shear-thinning property, and thus are easily injectable. Moreover, upon injection, they regain their mechanical property and structure preventing any leakage of drugs [124].

#### 4.2.2. Natural Polymer Based Self-Healing Hydrogels as Drug Delivery Platforms for Cancer Therapy

**Figure 8:**
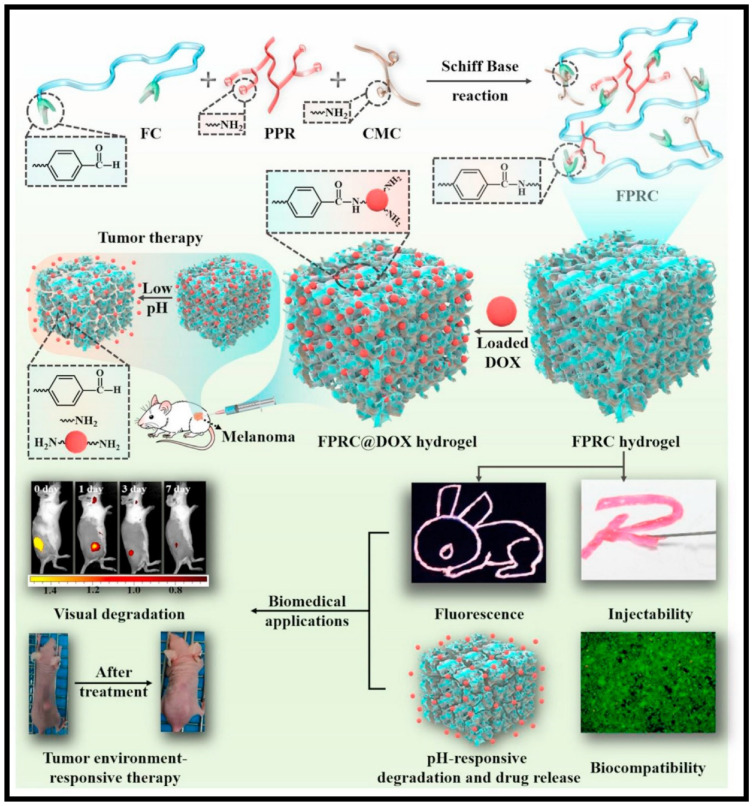
Schematic illustration on the method of fabrication of multifunctional FPRC Hydrogel and its multifunctional aspects like injectability, self-healing, photoluminescence, and pH-responsive degradation/drug release properties. (Reproduced with permission from Ref. [131]. Copyright 2020, Elsevier).

Natural polymers include those derived from plants, such as gum arabic, alginate, cellulose; as well as those derived from animals, such as gelatin, collagen, hyaluronic acid, chitosan, and others. These polymers were chemically modified to enable them with dynamic bonds which help them to acquire the required self-healing properties. Doxorubicin (Dox) is the most widely explored chemotherapeutic drug in terms of self-healing hydrogel applications. Qian et al. developed an injectable and self-healing hydrogel made using carboxyethyl-modified chitosan (CEC) and aldehyde modified hyaluronic acid (A-HA) through dynamic Schiff base bonds between amine groups on CEC and aldehyde groups on A-HA. They observed a pH-dependent Dox release, with an elevated release at acidic pH [129]. Yavvari et al. developed a non-cytotoxic and non-hemolytic chitosan-catechol-based self-healing hydrogel (CAT-Gel) assembled through Catechol-Fe (III) coordinative interactions. The hydrogel due to its amphiphilic nature was able to load both Dox and Docetaxel (DTX). The hydrogel was found to provide sequential and sustained release of the entrapped DOX, and DTX, and exhibited a synergistic therapeutic effect with increased median survival against murine lung and breast cancer models [130]. Recently, a multifunctional, injectable, self-healing, biodegradable citric acid-based scaffolds (FPRC Hydrogel) were developed using Schiff’s base reaction between F127–CHO (FC) [Pluoronic F127 with aldehyde group], PPR [red fluorescence-emissive polycitrate-polymine-rhodamine B polymer] and CMC [Carboxymethyl chitosan]. The scaffold exhibits multifunctional properties like thermo-sensitivity, injectability, self-healing, photoluminescence and pH-responsive degradation/drug release as shown in Figure 8. The scaffold showed pH-responsive Dox release and was found to have an enhanced therapeutic effect when compared to free Dox [131].

Natural products like guanosine and isoguanosine are currently explored as an anti-cancerous agents and self-healing hydrogels made using these natural products are currently being studied. Tang et al. developed self-healing supramolecular nucleoside hydrogel by simply mixing equimolar amounts of guanosine (G) and isoguanosine (isoG) in the presence of K^+^. However, this hydrogel was not found to be reasonably stable to be used in biomedical applications [132]. To further improve on this system, Zhao et al. developed a dual-functional supramolecular hydrogel (isoGBG hydrogel) using boric acids and diols derivatives of guanosine and isoguanosine through dynamic borate ester bonds. The isoGBG hydrogel was found to have excellent stability and self-healing properties. In both in vitro and in vivo studies, the hydrogel exhibits excellent anti-cancerous properties [133]. In another recent study, self-healing hydrogel was made using modified HA. HA was modified to have phenylboronic acid and dopamine moieties which upon interaction resulted in boronate ester bonds. Erlotinib (ERT) was encapsulated in PLGA microsphere, which was impregnated within the hydrogel for sustained and locoregional delivery. The microspheres impregnated hydrogel provided superior tumor-suppressive efficiencies compared to the other groups in A549 tumor-bearing mice [134]. Gelatin based self-healing hydrogels were explored for the delivery of 5-fluorouracil (5-FU) for cancer therapy. The self-healing mechanism is attributed to the reversible dimerization of ureidopyrimidinone (UPy) side chains present in gelatin (Gelatin-UPy) as well as through the coordination crosslinking by introducing Fe^3+^ in gelatin-UPy hydrogels [135]. An injectable self-healing hydrogel was made using Gum Arabic (GA) and Chitosan (CS) for the controlled release of nano curcumin as an anti-cancer treatment. Here, GA was modified with multi-aldehyde group (GAMA) to be reacted with the succinic anhydride-modified CS (SCS) resulting in the formation of a dynamic Schiff base linkage [136]. An injectable cellulose based self-healing hydrogel was developed using dynamic ketoester-type acyl-hydrazone bond. The hydrogel was found to have tunable mechanical property, pH responsiveness, injectability and biocompatibility. Furthermore, Dox was loaded into the hydrogel and pH-responsive release of the drug was demonstrated [137].

#### 4.2.3. Synthetic Polymer Based Self-Healing Hydrogels as Drug Delivery Platforms for Cancer Therapy

**Figure 9:**
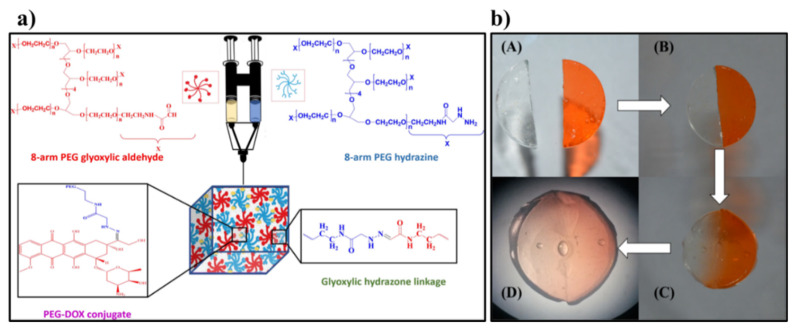
(**a**) Schematic representation of the steps involved in the fabrication of PEG Hydrogels from 8-arm PEG Glyoxylic Aldehyde, 8-arm PEG Hydrazine and 8-arm PEG Hydrazine partially modified with DOX, using hydrazone linkages. (**b**) Pictures showing the self-healing characteristic of hydrogels. (**A**) Freshly prepared hydrogel (with and without PEG−DOX loading). (**B**,**C**) Healing and diffusion of the conjugate at 0 and 2 h respectively. (**D**) Picture showing self-healed hydrogel with equilibrium drug diffusion after 6 h. (Reproduced with permission from Ref. [139]. Copyright 2019, The American Chemical Society).

Synthetic polymers are advantageous in terms of their highly controlled synthetic and processing procedure when compared to natural polymers, which suffer from their inherent batch-to-batch variations. Dox is again the most widely explored drug in terms of synthetic polymers based self-healing hydrogels. NIR/thermo-responsive self-healing hydrogel system comprising of polydopamine nanoparticles (PDA NPs) was developed. The self-healing hydrogels are made through dynamic covalent enamine bonds between the amino groups in polyetherimide (PEI) and the acetoacetate groups of the four-armed star-shaped poly(2-(dimethylamino) ethyl methacrylate-co-2-hydroxyethyl methacrylate) modified with tert-butyl acetoacetate (t-BAA), SP(DMAEMA-co-HEMA-AA) and PDA NPs. The injectable hydrogel upon intra-tumoral injections within 4T1 tumor in a mouse model showed improved Dox delivery within the tumor region as well as reduced adverse effects [138]. On similar lines, a self-healing hydrogel was synthesized through cross-linking reaction between 8-arm PEG glyoxylic aldehyde and 8-arm PEG hydrazine using glyoxylic hydrazone linkages, which is shown in Figure 9. Dox was covalently linked to the 8-arm PEG hydrazine in sub-stoichiometric ratios precedent to the hydrogel fabrication. A pH dependent enhanced release of Dox was observed in tumor pH condition when compared to physiological pH [139].

In another interesting approach, thermo-responsive self-healing hydrogels with aggregation-induced emission (AIE) property were developed using tetraphenylethylene (TPE) containing TPE-poly(*N*,*N*-dimethylacrylamide-stat-Diacetone acrylamide) [TPE-P(DMA-stat-DAA)] cross-linked by diacylhydrazide. The hydrogels were found to show an enhanced light emitting property above its lower critical solution temperature (LCST) based on the AIE property of the TPE unit. Moreover, the hydrogel was found to have pH-responsive Dox release, which can be used for anti-cancer therapy. These self-healing hydrogels could be powerful tools for localized drug delivery and real-time monitoring in cancer therapy [140]. An amphiphilic non-toxic pentablock terpolypeptide [PLys-b-(PHIS-co-PBLG)-PLys-b-(PHIS-co-PBLG)-b-PLys] was developed as a pH- and enzyme-responsive self-healing hydrogel for the delivery of Gemcitabine to treat pancreatic cancer. The hydrogel is easy to make and inject. Moreover, due to its pH-responsiveness, the hydrogel transforms into a liquid in the cancer tissue, mainly due to its lower pH, thus releasing the drug only within the cancer tissue [141]. Zhao et al. developed a photo-labile injectable self-healing hydrogel based on the hydrophobic interaction of a four-arms star polymer, poly (ethylene gly-col)-b-poly (g-o-nitrobenzyl-L-glutamate), a biocompatible material. Upon UV irradiation, the photolabile o-nitribenzyl ester group is cleaved, converting the hydrophobic group into hydrophilic ones resulting in the release of Dox, which promotes the apoptosis ratio of HeLa cells [142].

#### 4.2.4. Hybrid Polymeric Self-Healing Hydrogels as Drug Delivery Platforms for Anti-Cancer Therapy

Hybrid polymeric self-healing hydrogel includes those which have both the natural and synthetic polymers together in a single system to impart self-healing property. An injectable and pH-responsive self-healing hydrogel was developed through a dynamic covalent Schiff-base linkage between amine groups of N-carboxyethyl chitosan (CEC) and benzaldehyde groups from poly (ethylene glycol) (PEGDA). The hydrogel was found to have pH-dependent Dox release profile and was found to be effective against hepatocellular carcinoma [143]. On similar lines, another research group developed an injectable hydrogel with pH-sensitive and self-healing properties through Schiff base bonds formed between 4-arm PEG-benzaldehyde (4armPEGDA) and N-carboxyethyl chitosan (CEC). The hydrogel was found to have pH- responsive Dox release and was found to be effective against hepatocellular carcinoma cells both in the in vitro cell studies as well as in HepG2 nude mouse model [144]. A dynamic, pH-responsive, and biodegradable hydrazone linkages were formed between oxidized xanthan and 8-arm PEG hydrazine resulting in a self-healing hydrogel for the controlled release of Doxorubicin (Dox) [145]. Poudel et al. demonstrated the synthesis of self-healing supramolecular hydrogel system through host-guest interaction between the α-cyclodextrin (α- CD) and poly (ethylene glycol) (PEG) chains of the poly (ethylene glycol)-block-poly (lactic acid) (PEG-b-PLA) micelles. Dox loaded PEG-b-PLA micelles were used for making the hydrogel, and the released Dox showed improved performance against HeLa cells compared with the free Dox [146].

A self-healing thermosensitive gel was developed using Schiff’s base chemistry between dialdehyde-functionalized polyethylene glycol (DF-PEG) and β-glycerophosphate (GP) cross-linked chitosan (CS) hydrogels. Dox loaded self-healing thermosensitive gels showed an enhanced tumor inhibition rate (66.12%) than CS thermosensitive hydrogels (53.23%), upon intra-tumoral injection in Heps tumor-bearing mice [147]. In a recent study, An et al. reported a self-healing hydrogel based on the dynamic nature of acylhydrazone bonds between pectin aldehyde (pectin-CHO) and acylhydrazide functionalized polymer poly(*N*-isopropylacrylamide-stat-acylhydrazide) P(NIPAM-stat-AH). The hydrogel was found to load and release two drugs: doxorubicin hydrochloride (Dox∙HCl) and combretastatin A4 disodium phosphate (CA4) in a controlled manner that significantly improve the therapeutic effect as well as reduced the undesired toxic effects [148].

#### 4.2.5. Nanocomposite Self-Healing Hydrogels as Drug Delivery Platforms for Anti-Cancer Therapy

An injectable, self-healing hydrogel system with the magnetic property was developed by combining oxidized pectin, chitosan and γ-Fe_2_O_3_ nanoparticles. The self-healing property is obtained through the imine bond formed through Schiff’s base reaction between chitosan and the oxidized pectin. Magnetic hydrogel was made by dispersing γ-Fe_2_O_3_ nanoparticles of 250 nm in size on the surface of the hydrogel. The hydrogel was found to have good pH and thermo-responsive properties. Magnetic hysteresis loops of the hydrogel have S-shape over the applied magnetics and the saturation magnetization value of the hydrogel was about 4.86 emu/g. The hydrogel was found to have sustained release of 5-FU for more than 12 h in the in vitro drug release studies [149].

Self-healing hydrogels were designed to exhibit near-infrared laser (NIR)-triggered photothermal therapy. The hydrogel (CSMA/BPEI/BPEI-GO hydrogel) was synthesized using Schiff-base linkage between chondroitin sulfate multialdehyde (CSMA), branched polyethylenimine (BPEI) and BPEI conjugated graphene oxide (BPEI-GO). BPEI-GO doped in the hydrogel provides sustained drug delivery, and near-infrared laser (NIR)-triggered photothermal effect. The hydrogel also provides pronounced anticancer effects in a breast cancer postoperative recurrence prevention mice model. The Dox and photothermal therapy in CSMA/BPEI/BPEI-GO hydrogels group significantly reduced tumor recurrence when compared to other control groups [150].

Wang et al. developed a self-healing hydrogel system for an efficient synergistic magnetothermal-chemo-chemodynamic therapy for cancer. The hydrogel is made using Schiff base linkage between the benzaldehyde-functionalized pullulan (PULL-CHO) and chitosan-g-PEG (CS-g-PEG). The hyperthermic effect was induced by Rhodamine B isothiocyanate (RBITC)-labeled mesoporous silica nanospheres with Mn-Zn Ferrite (Mn_0.6_Zn_0.4_Fe_2_O_4_) nanoparticle core (MMSN-RBITC), which are well-dispersed into the hydrogel system. The nanoparticles also exhibited T2-weighted magnetic resonance imaging contrast enhancement. The Dox loaded self-healing hydrogel was found to impart excellent theragnostic property by providing tumor diagnosis and efficiently synergistic magnetothermal-chemo/chemodynamic therapy [151].

A multi-agent self-healing thermosensitive magnetic hydrogel comprising of dual drugs (Dox and Docetaxel) and magnetic nanoparticles-called as DDMH are explored for cancer therapy. The self-healing hydrogel is made using cheap and simple chitosan hydrogel cross-linked with telechelic difunctional poly-ethylene glycol (DF-PEG-DF). The hydrogel was found to exhibit good magnetic field responsive heat-inducing property, which could be used for magnetic field responsive delivery agents for the two drugs. It was found that DDMH has significant anti-tumor activity when compared to single drug loaded hydrogel and thus a potential multiagent co-delivery system for synergistic chemo-hyperthermic therapy for triple-negative breast cancer [128].

#### 4.2.6. Self-Healing Hydrogels as Drug Delivery Platforms for Antimicrobial Therapy

**Figure 10:**
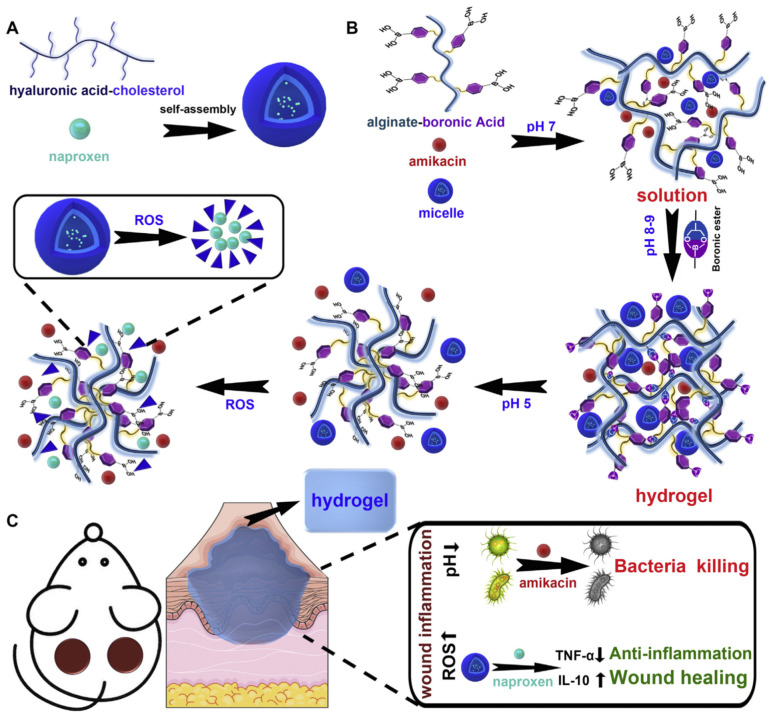
(**A**) Schematic representation of the fabrication of micelle. (**B**) Schematic representation of the synthesis route and drug release mechanisms for self-healing hydrogels. (**C**) Schematic illustration of antibacterial and wound healing mechanisms. (Reproduced with permission from Ref. [152]. Copyright 2020, Elsevier).

Self-healing hydrogels are typically administered as injectable hydrogels, which could be squeezed while injecting them due to its shear-thinning property and get gelled upon reaching the target site. Furthermore, the lifespan of the hydrogel can be enhanced owing to its self-healing property, which repairs the damaged region quickly without the need for frequent administration. Recently, an inflammation region responsive self-healing hydrogel system comprising of antibiotic amikacin and micelles loaded with anti-inflammatory drug naproxen was reported, as shown in Figure 10. The hydrogel is made by grafting phenylboronic acid to the side chain of the alginate, resulting in a smart pH and reactive oxygen species (ROS)-responsive injectable hydrogel. The hydrogel is found to be biocompatible, showed good antibacterial and anti-inflammatory properties [152].

Chitosan, known for its antimicrobial property is widely explored as a natural polymer to impart self-healing property for this application. Chitosan and amino acid (acryloyl-phenylalanine) based self-healing hydrogels were fabricated through in situ polymerization using ammonium per sulfate as a redox initiator. The hydrogel exhibited a controlled release of the loaded antimicrobial agent, levofloxacin, whereby 70% of the loaded drug is released in 60 h [153]. Sharma et al. developed chitosan-cuminaldehyde self-healing hydrogel by forming a dynamic imine bond between chitosan and cuminaldehyde. The hydrogel showed excellent self-healing properties and controlled release of levofloxacin for 90 h [154]. In another study, levofloxacin was loaded into a self-healing hydrogel fabricated through in situ free radical polymerization of guar gum-graft-acrylic acid (GG-PAA) in presence of L-Alanine as the crosslinker. The hydrogel exhibited sustained release of levofloxacin at physiological pH and temperature for up to 130 h [155]. In a different approach, self-healing hydrogel was used to deliver zinc ions. An injectable self-healing hydrogel was developed using carboxymethyl chitosan (CMCh) cross-linked by zinc ions (Zn^2+^). The hydrogel exhibited self-healing property and was found to be effective against both *Staphylococcus aureus* (*S. aureus*) and *Escherichia coli* (*E. coli*) [51]. Another boronic ester dynamic covalent bond based self-healing hydrogel using phenyl boronic acid-modified hyaluronic acid (HA–PBA) and plant-derived polyphenol-tannic acid (TA) was developed by Shi et al. They have also used TA for the synthesis of silver nanoparticles within the hydrogel, which have antibacterial properties. The hydrogel was found to have dual (pH- and reactive oxygen species (ROS)) responsive properties as well as enhanced antioxidant properties [156].

A local drug delivery system for bone tuberculosis therapy was fabricated using a self-healing hydrogel made using PLGA-PEG-PLGA co-polymeric system, comprising of a derivative of isoniazid (DINH) loaded liposomes. The hydrogel was found to have excellent self-healing properties. It was also found to have initial burst release (reaching an effective inhibitory concentration) followed by steady-state release in the synovial fluid through in vivo micro-dialysis studies [157]. In another advancement for the treatment of acute bacterial rhinosinusitis (ABRS), polyethylene glycol-based self-healing hydrogel comprising of Clarithromycin liposomes (CAM-Lips@Hydrogel) or Clarithromycin and Budesonide loaded liposomes (CAM+BUD-Lips@Hydrogel) was developed. The hydrogel is formed through a high affinity and dynamic reversible coordination bond between the thiol group of 4-arm-PEG-SH and silver ions. Moreover, in vivo studies indicate that the hydrogels not only play an effective role as an anti-bacterial, but also inhibits the inflammatory response of local sinus mucosa both in a single or combined load [158].

An antifouling and self-healable poly (dimethyl siloxane) (PDMS) based hydrogel was reported for contact lenses application. The hydrogel was made using Schiff’s base reaction between amine functionalized PDMS based polyzwitterionic polymersomes and polyethylene glycol dialdehyde (PEG-DA). This hydrogel exhibited sustained release of the entrapped curcumin for more than 72 h. It was found that the curcumin loaded hydrogel was effective against both gram-negative (*E. coli*) and gram positive (*S. aureus*) bacteria. This self-healing hydrogel system can be a potential system for therapeutic applications in several eye diseases [159]. Liang et al. developed hyaluronic acid-g-dopamine based adhesive self-healing hemostatic antioxidant conductive photothermal antibacterial hydrogels. The hydrogel is impregnated with polydopamine coated reduced graphene oxide to impart conducting property to it. The hydrogel was cross-linked with an oxidative coupling reaction via their catechol group by using H_2_O_2_/HRP as an initiator system. Furthermore, the hydrogel showed sustained doxycycline release and significantly enhance vascularization by upregulating growth factor expression of CD31 [160].

#### 4.2.7. Self-Healing Hydrogels as Drug Delivery Platforms for other Applications

Guanosine based self-healing hydrogel was reported for the on-demand delivery of Acyclovir, an antiviral agent. Guanosine-quartet Na^+^-borate based hydrogel was synthesized through dynamic guanosine–borate diester bond and intertwined by G4-nanofibres formed by π–π stacking of G4-quartets stabilized by Na^+^ ions. Unlike conventional hydrogels, this hydrogel was found to have excellent self-healing and on-demand drug release behavior without early burst release [161]. Dexamethasone, a glucocorticoid used as anti-inflammatory and immunosuppressant molecule is explored with self-healing hydrogels-based drug delivery systems. Yu et al. reported a novel hydrogel synthesized through a non-covalent host-guest interaction between β-cyclodextrin modified hyaluronic acid (HA-CD) and adamantane modified 4-arm-PEG (4-arm-PEG-Ad). Dexamethasone was found to be loaded within the hydrophobic pockets of β-CDs. The hydrogel was found to exhibit significantly enhanced therapeutic effect of dexamethasone in burn wound healing [162]. In another study, super-stretchable, self-healing and injectable hydrogel was developed through host-guest interactions between octa-cyclodextrin polyhedral oligomeric silsesquioxane (OCDPOSS) and acrylamide-modified adamantane (Ad-AAm) under UV irradiation. The hydrogel showed controlled release of Dexamethasone, leading to the long-term release of the drug at injected locations [163].

Basu et al. developed DNA-based hydrogel crosslinked with oxidized alginate (OA) through dynamic imine linkages. Silicate nanoparticles provide electrostatic interaction with the negatively charged DNA strands, resulting in physical crosslinking points. The hydrogel was shown to provide sustained simvastatin release for a week. [164]. In case of treatment for osteoarthritis, colchicine loaded mesoporous silica nanoparticles/hydrogel composite in a cotton patch was developed as a transdermal patch. The hydrogel was synthesized by reacting carboxyethyl chitosan and oxidized pullulan. The patches when implanted on a mono-iodoacetate (MIA)-induced rat osteoarthritis model showed improved locomotor activity, glutathione blood level, and a significant decrease in levels of malondialdehyde, nitric oxide, TNF-α, and COX-2 [165]. In another interesting approach, Appel et al. synthesized shear-thinning, self-healable injectable hydrogels by utilizing hydrophobic interactions between hydroxy-propyl-methyl cellulose derivatives (HPMC-x) and PEG-b-PLA nanoparticles. The hydrogels containing PEG-b-PLA NPs enables dual loading of a hydrophobic molecule into the PEG-b-PLA NPs and a second hydrophilic molecule into the gel structure. The hydrogel showed self-healing property with the possibility of dual drug delivery applications [166].

### 4.3. Effect of Self-Healing Properties on Cells

**Figure 11:**
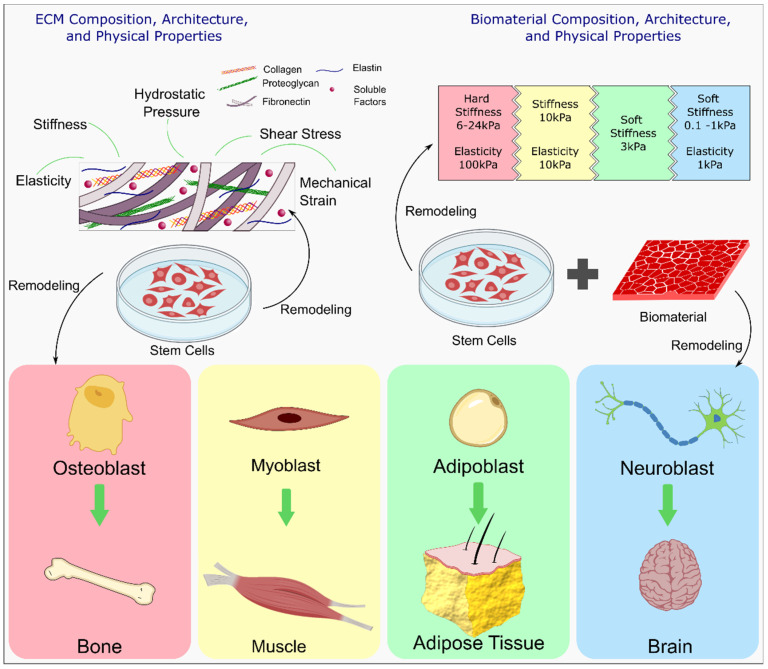
Schematic of the interaction between stem cells and ECM and its effect on ECM properties along with pathways that determine cell fate. The different biomechanical properties of the ECM promote differentiation of cells towards tissue specific lineages and is dependent on the composition, architecture, microstructure, and the various cues within the ECM (Modified and reproduced with permission from Ref. [171] under Creative Commons Attribution License (CC BY)).

Cell therapy is a viable approach and strategy to treat a plethora of diseases and aid in tissue regeneration. Engineered hydrogels are highly desirable in cell therapy due to their tunable properties and their resemblance to the natural 3D structure of the extracellular matrix (ECM). Cell encapsulation within hydrogels as a vehicle for cell delivery is garnering attention because hydrogels can be tuned to have desired tissue-specific biochemical, biophysical, and biomechanical properties. They can be tuned to control cellular functions such as migration, cell adhesion, proliferation, and differentiation [167]. Additionally, for their use as cell delivery modality, hydrogels must exhibit good biocompatibility and biodegradability [168]. A significant challenge in cell therapy is the designing of ideal cell carriers that can deliver cells to the target site efficiently while avoiding challenges such as limited cell retention at the site and low rates of cell survivability that are typical of traditional cell-based therapies [46]. However, traditional hydrogels composed of static networks with low mechanical strength, are fragile, prone to breakage or loss of properties and lack of required dynamics for cell expansion and matrix remodeling. This limits their feasibility in tissue regeneration [62,169]. The flexibility and permeability possessed by self-healing hydrogels enhances nutrient exchange and increases the viability of cells. Additionally, they provide the chemical and physical dynamism required to control cell growth, behavior, and fate [62,170] and mitigate the shortcomings of traditional hydrogels. Figure 11 highlights the composition, architecture, and physical properties of the ECM and the factors that contribute to cell fate and ECM remodeling.

Due to their hydrophilic and injectable nature, hydrogels are favored as cell carriers. A key aim of cell encapsulation within a hydrogel is to protect the cells while in transition to the site of injury [170]. Self-healing hydrogels are able to intrinsically and automatically heal damages and restore their shape and properties. This ability is based on reversible or dynamic crosslinking chemistries [8], thereby giving protection to the encapsulated cells through the dissipation of shear and compressive forces [167]. Extrusion based 3D bioprinting relies on shear-thinning properties of hydrogels where the viscosity of the hydrogel reduces under an applied shear and could prevent sedimentation of cells [172,173]. While static hydrogels play a minimalistic role in regulating and guiding cellular behavior and cell fate within the microenvironment, self-healing hydrogels have a more influential effect effects on these aspects.

#### 4.3.1. Maintaining Cell Stemness

**Figure 12:**
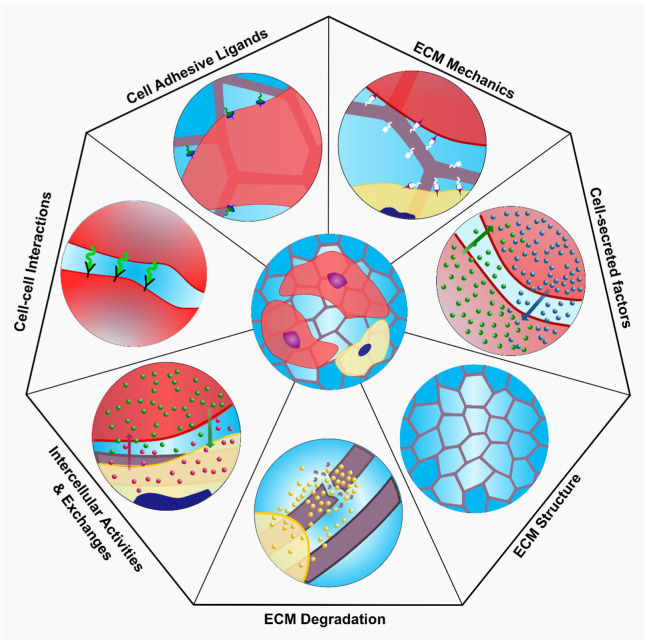
Schematic of interactions known to regulate stem cell fate within ECM structure.

Hydrogels are employed as cell carriers for stem cell therapy, in which stem cells expanded in vitro are encapsulated within the hydrogel structure and implanted at the site of injury. While stem cell therapies involving direct injection of cells at the site of injury face low retention, low survivability and uncontrolled differentiation, their incorporation within a hydrogel scaffold has mitigated these challenges [174]. However, a major challenge in clinical translation of tissue engineering and regenerative medicine involving live cells is the requirement of large quantities of cells [175]. According to Cheung et al. stem cells are proposed to exist in a proliferative or quiescent state [176]. A stem cell encompasses the capability for self-renewal and differentiation through a balance between self-renewing proliferation, differentiate into tissue-specific cell type and quiescence via microenvironment interactions [177]. The microenvironment interactions are governed via ECM mechanics, engineered matrix degradation, cell-adhesive ligands, ECM microstructure, cell-cell interactions, cell-secreted factors, heterologous cell interactions (Figure 12). Maintaining stemness within the hydrogel is necessary for delivering cells for therapeutic purposes, which may include expansion of stem cells or drug delivery at the site of tissue regeneration and tissue repair. Cells in proliferative state can be used in regenerative medicine application. However, long-term culturing of stem cells for disease modelling and drug screening platform may necessitate stem cell quiescence. While the structure of permanently cross-linked hydrogels may provide an inadequate niche for maintaining stemness, the advances in self-healing hydrogels coupled with design principles of stem cell niche can be employed to prompt desired stem cell phenotype for tissue specific application. While inefficient scalable production of high-quality stem cells remains a challenge, the use of adaptable hydrogels may provide a viable solution towards the generation of scalable and adaptable platforms that improve stem cell production efficiency. In the following subsections, we will discuss the properties of engineered hydrogels that recapitulate the stem cell niche environment.

#### 4.3.2. ECM Mechanics

The ECM is a 3D macromolecular network composed of proteins, polysaccharides, elastin, water, and several other glycoproteins that provide a physical scaffolding for cells and is also responsible for various biochemical and biomechanical cues required for cellular adhesion, differentiation, morphogenesis, and homeostasis [178,179]. Due to the mechanical coupling between cells and ECM, the mechanical properties of the ECM has a profound effect on altering, regulating and modulating cell spreading, cell shape, intercellular communication, and signaling pathways [180]. ECM stiffness is also recognised to play an important role in the directional differentiation of stem cells [165,176]. Neural stem cells are capable of differentiating into neurons and glial cells for neural regeneration; however, their fates are modulated by the stiffness of the scaffold. In hydrogels with stiffness in the range of 0.1–1 kPa, neuroblasts are prone to neural differentiation while on stiffer materials in the range of 7–10 kPa, they are prone to glial differentiation [181,182] as shown in Figure 11. Similarly, load-bearing tissues are mechanically stronger and denser than non-load bearing tissues and therefore, highly swollen hydrogels are inadequate for use in bone tissue engineering application as they do not possess the mechanical integrity to support large load. ECM porosity also plays a critical role in materials mechanical properties and controls permeability of cells and diffusion of nutrients in and waste out of a scaffold. Zhang et al. developed a tough composite hydrogel composed of laser ablated porous PLGA skeleton grafted with hydrophobic Polycaprolactone (PCL). Owing to the permeability of self-healing hydrogels, the authors observed efficient diffusion of oxygen and nutrients resulting in high survivability of cells after 7 days in culture [62]. Notably, the dynamic crosstalk between the ECM and cells enables remodeling of the ECM composition and architecture through the secretion of ECM structural components and matrix metalloproteinases. Creating a proper milieu with appropriate biological and mechanical cues that promote mechano-sensing and mechano-transduction signals to guide stem cell activity and fate remains a challenge when creating biomaterials as scaffolds for cell encapsulation [183]. Additionally, it is important to design self-healing hydrogel with tunable stiffness that allow incorporation of necessary bioactive molecules such as transforming growth factor β (TGF-β) which is known to enhance ECM production [184].

#### 4.3.3. Cell-Adhesive Ligands

Cells are attached to the ECM network via transmembrane proteins called integrins. Integrins are cell adhesion receptors that bind to specific cell-adhesive ligands embedded within ECM proteins and connecting to the network via intracellular actin cytoskeleton [180]. The tripeptide arginine-glycine-aspartic acid (RGD) found within ECM proteins bind with several integrin dimers, triggering cell adhesion and elicit cell responses [185,186]. Additionally, native ECM is a mixture of proteins and polysaccharides, thus recent works in self-healing hydrogels have seen the incorporation of collagen, gelatin, alginate and chitosan or their composites [59,181,187,188]. There has also been significant research on the use of peptides in the generation of adaptable hydrogels for tissue regeneration [11,189,190]. This is attributed to take advantage of the natural bioactive molecules present in these hydrogels and in the case of peptide-based hydrogels to promote cell adhesion and cell proliferation moieties.

#### 4.3.4. Cell-Cell Interactions

**Figure 13:**
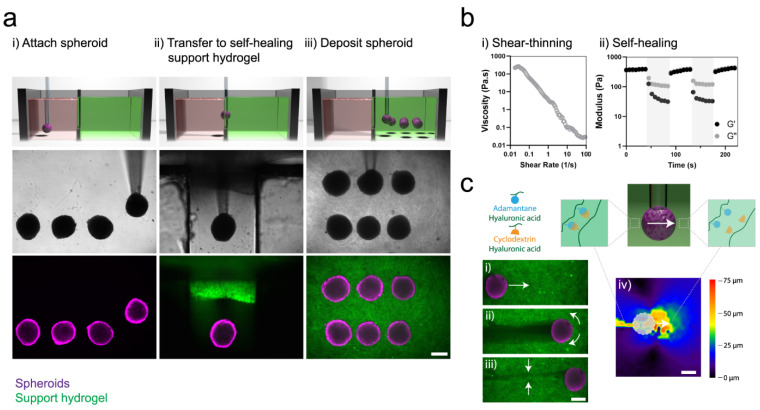
(**a**) Schematic, brightfield and fluorescent images (top, middle, bottom respectively) of (**i**) formation of spheroid loaded with MSC in support bath, (**ii**) transfer of spheroid to self-healing support hydrogel followed by (**iii**) deposition of spheroid within support hydrogel. (**b**) Rheological characterization and demonstration of (**i**) shear thinning properties of the guest-host support self-healing hydrogel. (**ii**) self-healing properties: storage modulus and loss modulus. (**c**) demonstration of reversible interactions between spheroid and support hydrogel. (**i**,**ii**) yielding of the support hydrogel post spheroid translation and the resulting (**iii**) rapid healing of the support hydrogel after spheroid translation. (**iv**) mapping the displacement of the support hydrogel highlighting the local motion of the hydrogel around the spheroid. (Reproduced with permission from Ref. [193] under Creative Commons Attribution License (CC BY)).

The natural ECM is a complex three-dimensional network, and it is in this milieu that most cellular interactions take place. While most of the experimental evidence regarding cell-cell and cell-ECM interactions were conducted on 2D planar surfaces, these surfaces do not recapitulate the biological processes and biomimetic properties of 3D structures. Spheroids and organoids can be used as models to study the niche environment however, they are limited by complexity, throughput, and reproducibility [191]. On the other hand, extrusion-based 3D bioprinting is an emerging technology with the capability to generate complex 3D networks. For example, cardiovascular diseases involve pathological remodeling by fibrosis due to increased proliferation of cardiac fibroblast and ECM deposition [192]. Healthy and functional cardiac tissue requires cohesive cell-cell interactions to support the action potential propagation and synchronized contraction. Cardiac fibroblasts are unable to generate an action potential and along with excess deposition of collagen post-cardiac disease, there is a reduction in intercellular connectivity resulting in reduced contractility and electrical synchronization [192]. Recently, Daly et al. reported a method to translate spheroids through a shear-thinning self-healing hydrogel that can hold the spheroid in 3D space allowing for the generation of high cell density microtissue with increased cell-cell interaction (Figure 13) [193]. In their report, they mixed iPSC-derived cardiomyocytes and cardiac fibroblasts at various ratios to study the interactions with favorable results. Self-healing hydrogels enabled the authors to maintain spheroid shape however, it is established that due to lack of vasculature to deliver oxygen and essential nutrients, the current model may not recapitulate the in vivo 3D architecture and further studies are required to improve such models.

#### 4.3.5. Cell-Secreted Factors

Paracrine signaling by stem cells plays a vital role in the effective implementation of tissue regenerative therapies [194]. However, the exact mechanisms through which stem cells repair facilitates healing and the role self-healing hydrogels in aiding this mechanism requires further investigation. Thomas et al. studied the response of paracrine factors secreted by implanted cells by encapsulating mesenchymal stem cells within an injectable self-healing hybrid hydrogel incorporated with a Schiff base linkage between the aldehyde groups on carboxymethyl cellulose dialdehyde (CMC-D) and amino groups on carboxymethyl chitosan (CMCh). Their study indicated that the scaffold promoted chemotactic, proliferative, and wound-healing response of cells to paracrine factors in vitro, however, the efficacy needs to be investigated in vivo [26,59].

#### 4.3.6. Cell Encapsulation and Stem Cell Differentiation

Wei et al. developed a self-healing hydrogel made of gelatin cross-linked by oxidized dextran as an injectable carrier of endothelial progenitors to study vascular morphogenesis in vivo [64]. They observed that the hydrogel protects cells from shear forces generated during ejection from a syringe and allows controllable spatial placement of cells and this was attributed to dynamic imine cross-links between gelatin and oxidized dextran [64]. Lu et al. developed an injectable self-healing hydrogel based on chondroitin sulfate multiple aldehydes (CSMA) and N-succinyl-chitosan (SC). They were able to control the hydrogel stiffness, water content and gelation kinetics by varying the ratio of CSMA to SC. By encapsulating HeLa cells within the hydrogel, they observed good proliferation and migration of the cells within the network, however, in vivo studies were only performed to study degradation of the injected hydrogel without the presence of cells. Inflammation response after 3 weeks of injection was observed in the animal model which diminished within 7 weeks. However, cell differentiation, proliferation and wound healing at the site of induced injury requires further investigation [46].

It is well established that the ECM influences and regulates stem cell differentiation through a combination of growth factors, matrices, and forces [195,196,197]. Hence, there is considerable interest in the development of self-healing hydrogels to promote tissue-lineage specific differentiation. In their study of an injectable carboxyethyl chitin/dibenzaldehyde-terminated poly(ethylene glycol) (CECT-ADH/PEG-DA) hydrogels’ ability to support multipotent differentiation, Yang et al. embedded rat bone marrow derived MSCs (rMSCs) within the hydrogel incubated in osteogenic, adipogenic, and chondrogenic media for 7 days and measured the gene expression levels of the differentiation markers [198]. Their study reported that the self-healing hydrogels were able to maintain their stemness including the capability to proliferate and differentiate into multiple cell lines and this was attributed to the chitin hydrogels efficient enzymatic degradability [198]. However, whether this capability is possible under in vivo and biological environments needs further investigation.

### 4.4. Self-Healing Hydrogels in Cartilage and Bone Tissue Engineering

Owing to their dynamic and reversible properties, self-healing hydrogels are predominantly desirable in tissue engineering field. These hydrogels promote ECM deposition as well as help in the maintenance of cellular phenotype due to their dynamic 3D microenvironment. Bone is a hard tissue that forms the vertebrate skeleton in humans and animals also, it is one of the most transplanted tissues in the world. Apart from biological and structural features, the suitable mechanical characteristic (bone and muscle), electrical cues (cardiac, nerve) and quick recovery capabilities from the reversible self-healing hydrogel networks are crucial for hard tissue engineering applications [3,10,11,199,200]. Based on the above requirements, engineered hydrogels with the combination of mechanical toughness, electroactivity and self-healable properties have attracted a great deal of attention in tissue engineering applications. These hydrogels, due to their pre-gelling fluidity can fill asymmetrical tissue defects which evade the pre-shaping and invasive surgery. For regenerative engineering applications, self-healing hydrogels with dynamic mechanical microenvironment for cells with adaptable properties using reversible linkages were recently reviewed by Tong et al. [170]. Significant aspects to design the adaptable hydrogels are their fast stress relaxation and easy remodeling capacity. Different reversible linkages could be implemented by analyzing the gelation rate and other physiological conditions such as pH, redox etc. Some specific tissue engineering applications with targeted cell differentiation requires deliberately regulated fast stress relaxation [170].

**Figure 14:**
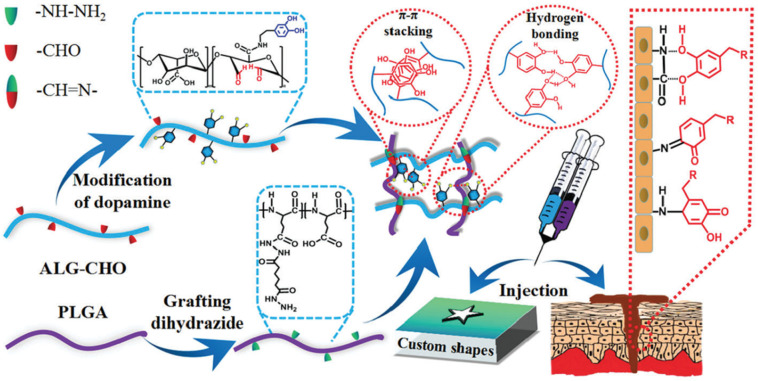
Schematic representation of the development of mussel-inspired PLGA/ALG–CHO–Catechol adhesive injectable self-healing hydrogels. (Reproduced with permission from Ref. [205]. Copyright 2018, The Royal Society of Chemistry).

Highly functional self-healing hydrogel with injectable, native tissue mimetic and biocompatible properties was developed by combining supramolecular chemistry and hydrogels [3]. This was achieved through a reversibly crosslinked hydrogel network. Saunders et al. recently reviewed the role of self-healing hydrogels in the field of tissue engineering [3]. Tissue engineering applications of supramolecular self-healing hydrogels have been developed by including novel chemistry and ECM properties into supramolecular hydrogel design that can favorably combine the properties of native tissue architecture and provide cell delivery and signaling cues. In the development of self-healing supramolecular hydrogels, peptides are good option as building blocks because of their intrinsic bioactivity and biocompatibility. These hydrogels have also been used in the regeneration of hard tissues like bone [10,201,202,203], dental [204] and cartilage [3,199,203] which are a promising and novel strategy for hard tissue regeneration and treatment. Mussel-inspired injectable hydrogels with good mechanical, self-healing, adhesive, and hemostatic properties were prepared via the Schiff base reaction [205]. An adhesive motif, dopamine was grafted on the aldehyde-modified alginate backbones. Further dual-functionalized alginate (catechol- and aldehyde-modified alginate, ALG–CHO–Catechol) and hydrazide-modified poly(L-glutamic acid) (PLGA–ADH) were crosslinked to form PLGA/ALG–CHO–Catechol self-healing injectable hydrogel system (Figure 14). 3D hydrogel network was formed by self-crosslinking when PLGA–ADH and ALG–CHO– Catechol were mixed together, permitting the defect site to be entirely matched and filled. Compared to oxidized ALG–CHO–Catechol hydrogels and PLGA/ALG–CHO hydrogels, PLGA/ALG–CHO–Catechol system showed notably improved properties in gelation time, mechanical behavior, and adhesive properties. Furthermore, this system showed good self-healing capability, cytocompatibility, bio-adhesion, and hemostatic performances. This hydrogel system showed the promising capacity for bone defect fixation with good hemostatic performance [205].

Similarly, multi-functional hydrogel with self-healing, adhesion, cytocompatibility, blood cell coagulation ability and hemocompatibility was developed using catechol-conjugated chitosan (CHI-C) for the treatment of bone bleeding and bone defect [206]. The idea behind this hydrogel development was as follows: in the area of bone defects and irregular bleeding sites, this hydrogel can be injected to fill the defects fully and sticks to the bleeding sites strongly. Then, it can self-heal rapidly within 2 min in the presence of body fluids. The results of this hydrogel application in rabbit ilium bone defect model revealed quick hemostasis and very less blood loss compared to untreated injuries. Furthermore, it helped in the disappearance of the bone defect and did not affect bone regeneration. In order to increase the strength and mechanical tunability of chondroitin sulfate (ChS) hydrogels, Diels Alder (DA) click chemistry as well as dynamic acylhydrazone bond cross-linking was employed during the preparation of the hydrogel [207]. The injectability and self-healing capability of the hydrogel were provided through dynamic acylhydrazone bond whereas in vivo stabilization of covalent crosslinking and modulation was achieved through DA click chemistry. Compared to single crosslinking thorough either DA click chemistry or acylhydrazone bonds combined strategy showed higher tunability in viscoelastic and rheological properties, injectability, self-healing, swelling and degradation behavior. In vivo studies revealed good tissue adhesiveness, improved viability, and decreased apoptosis of rat MSCs. In addition, new bone tissue was spotted on the cranial bone defect site where bone morphogenetic protein 4 (BMP-4) loaded hydrogel scaffolds were used. Hence, this hydrogel scaffold with injectability, self-healing ability and cytocompatibility with tunable properties can be an advantage for bone tissue regeneration.

Dynamic metal-ligand coordination chemistry was established for the development of silk fibroin (SF)-based hydrogels using SF microfibers (mSF) and a polysaccharide binder under physiological conditions [208]. This approach was developed to overcome issues such as lack of reversible crosslinking, problems associated with controlled gelation time and the usage of non-physiological conditions. For filling irregular shapes in tissue defects, this SF-based hydrogel showed self-healing and shear-thinning properties. In order to accelerate bone growth in cranial defects, calcium phosphate was coated onto mSF by biomineralization approach and reversible crosslinking was obtained through chelation of polysaccharide’s biphosphonate ligands. Acrylamide groups of the polysaccharide were photopolymerized to provide dually crosslinked (DC) robust hydrogel. These injectable and photopolymerized SF-based self-healing hydrogels can be a promising candidate for bone regeneration with personalized shape. Gelatin hydrogels were crosslinked through host-guest complexation and additionally reinforced with limited chemical cross-linkers to produce highly stable and distinctive cell-infiltratable and injectable (Ci-I) hydrogels [209]. Ci-I hydrogel was able to encapsulate cells and drugs which allows the endogenous cells in the curing course and facilitate continuous delivery of small drugs which reduced the risk of adverse effects due to drug overdose.

**Figure 15:**
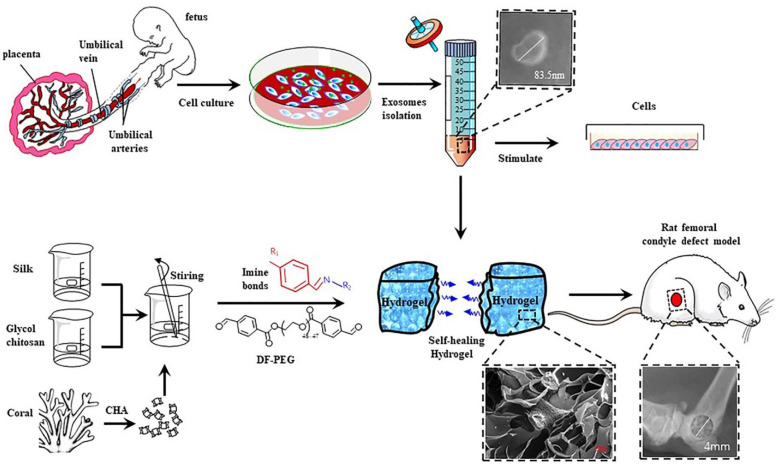
Schematic representation of the hucMSC-derived exosomes isolation and synthesis of CHA/SF/GCS/DF-PEG hydrogel for bone defect treatment in rat bone defect model. (Reproduced with permission from Ref. [210]) under Creative Commons Attribution License (CC BY)).

In order to improve and expand the functionalities of hydrogels, loading them with bioactive components is a significant technique, although this comes with a challenge of spatio-temporal precision. However, at the same time it is a very challenging approach for the precise delivery of these components in specific locations. Encapsulation of drugs and mesenchymal stem cells (MSCs) averted the reduction of bone mineral density (BMD) but stimulated the in situ bone regeneration in an animal model. This study revealed the ability of these injectable hydrogels as an active carrier for drugs and biomolecules to treat defective anatomical sites in a deep and enclosed area through this minimally invasive process. A new type of composite self-healing hydrogel containing human umbilical cord mesenchymal stem cells (hucMSC) was prepared using coralline hydroxyapatite (CHA)/silk fibroin (SF)/glycol chitosan (GCS)/difunctionalized polyethylene glycol (DF-PEG) for bone defect treatment (Figure 15) [210]. The results revealed the precipitation of CHA crystals similar to natural bone minerals, higher self-healing capacity and better surface morphology. The biological analysis through in vivo studies proved the effective bone healing ability of the hydrogel as well as its non-toxicity and biocompatibility towards rat bone defect. In the hucMSC group, new bone formation and higher microvessel density were observed.

Multifunctional injectable, self-healing, and antibacterial hydrogel drug delivery system for osteoporotic fractures and bone marrow cavity treatment was developed using PEG hydrogel incorporated with adhesive liposomes (A-LIP) loaded with BMP-2 [211]. This adhesive lipo-hydrogel (A-LIP-PEG) can be injected into a defective site where this hydrogel can release A-LIP that can adhere to the bone defect and promote bone regeneration. A-LIP-PEG hydrogel contains thiolated polyethylene (SH-PEG) and Ag+, which provide injectability and self-healing properties whereas the presence of Ag+ inhibits bacterial growth. A-LIP-PEG hydrogel significantly improves osteogenic differentiation and tissue adhesion in vivo. This A-LIP-PEG can be used in various other clinical applications such as skin wound regeneration, infections due to orthopedic implants and focal treatment of cancer [211]. Similarly, non-invasive, self-healing and injectable nanocomposite hydrogels were developed to improve the present orthopedic treatment approach. In this report, the gelation process of the polysaccharide matrices was accelerated through a hydrogen bond with the added LAPONITE^®^ (LAP) nanoplatelets [212]. This nanocomposite hydrogel showed better injectability, self-healing, enhanced mechanical and rheological properties. Furthermore, the LAP nanoplatelets showed strong static binding towards bone morphogenetic protein-2 (BMP-2) and form stable LAP@BMP-2 complexes which prolonged the release period. Additionally, both in vitro and in vivo studies revealed that this complex hydrogel system improved cell spreading, proliferation and osteogenesis. This study provides a platform for using self-healing hydrogels for protein therapeutics and non-invasive bone repair.

**Figure 16:**
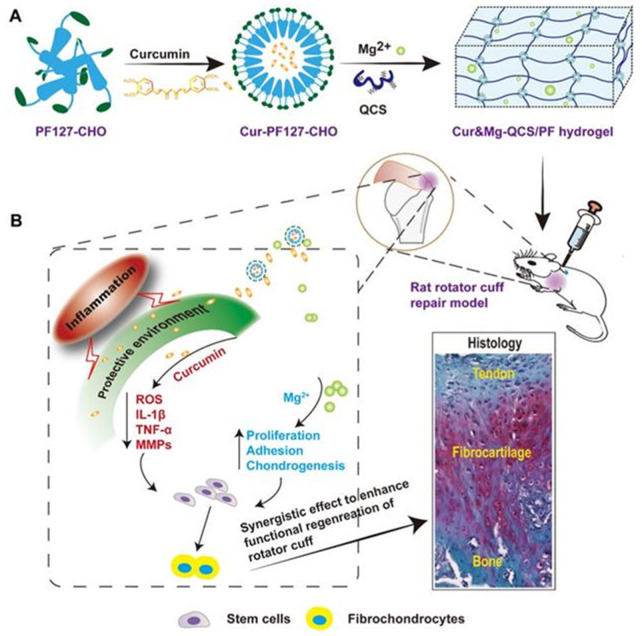
Schematic representation of (**A)** Cur&Mg-QCS/PF hydrogel preparation, (**B**) functional mechanism of Cur&Mg-QCS/PF hydrogel for the repair of rotator cuff injury. (Reproduced with permission from Ref. [213] under Creative Commons Attribution License (CC BY)).

Very recently, injectable and self-healing hydrogel comprising Mg^2+^/curcumin was used to treat rotator cuff injury [213]. Starting materials were quaternized chitosan (QCS) and Pluronic^®^F127 (PF127-CHO). Curcumin was added to PF127-CHO to produce Cur-PF127-CHO. Hydrogels (Cur&Mg-QCS/PF) were produced by dynamic Schiff base bonding between the amino groups of QCS and aldehyde groups of PF127-CHO/Cur-PF127-CHO with or without MgCl_2_⋅6H_2_O. Tendon-to-bone healing was achieved through the anti-inflammatory and pro-differentiation effects instigated via the sustained release of Mg^2+^/curcumin from the hydrogel. Results from this study revealed that these hydrogels possessed rapid self-healing, biocompatibility, better adhesiveness, and injectability (Figure 16). Tendon-to-bone healing by this composite hydrogel was confirmed through biomechanical and histological analysis of a rat model at 8 weeks after surgery. Similarly, *N*-[3-(4-hydroxyphenyl)propanamido] chitosan and a difunctional PF127 crosslinker have undergone a reaction to develop the phenolic-chitosan core-shell self-healing hydrogel (CPF) with fast gelling, thermoresponsive characters, fast adhesiveness, strong binding, and high water content (96.5 wt%) for future biomedical application with multifunctional features [214].

Another interesting nature of the supramolecular interactions in hydrogels is their self-integration property. This property may deliver an answer to the regeneration of tissue complexes with biocompatible and biodegradable properties. Discrete hydrogels with respective cell types and signaling molecules can be unified into a construct where different tissue constituents can be regenerated in the respective regions but unified at their interfaces. For example, in a recent report, a quadruple hydrogen-bond array Ureido-pyrimidinone (UPy) was used as a multiple hydrogen-bond unit and dextran (DEX) was used as polymer to achieve the aforementioned goal [199]. In this system, many UPy units were grafted onto DEX using the hydroxyl groups of DEX to form UPy-bearing polymers to understand the regeneration of cartilage–bone tissue complex in vivo. This injectable (shear-thinning) and self-integrating hydrogel with selected cells and biomolecules were used to regenerate cartilage-bone tissue complex in a subcutaneous implantation model. This study proves the multi-tissue complex engineering approach which can be used for other tissue complexes by integrating different cells and biomolecules [199].

Another interesting application of self-healing hydrogels are their attractive properties that can be used for 3D printing to develop scaffold for bone tissue engineering. 3D printed self-healing hydrogels for the development of soft and dynamic scaffolds will allow us to enhance the properties and resilience of the printed scaffolds. However, the studies on 3D printed self-healing hydrogel scaffolds are in its initial phase with limited reports. By employing commercially available materials and digital light processing unit, PVA based self-healable 3D printed hydrogel was fabricated by a research group [215]. Self-repairing in these hydrogels was enabled by a semi-interpenetrated polymeric network exclusive to any external trigger, and the repair occurred independently at room temperature. These samples recovered well to gain 72% of their initial strength and withstand deformation. This method enabled to use 3D printed self-healing hydrogels for various applications. 3D printed self-healing microporous hydrogels were prepared through crosslinking of poly(*N*-hydroxyethyl acrylamide-co-methyl vinyl ketone) (PHEAA-co-PMVK) by bifunctional hydroxylamine bond formation [216]. After the post processing, the mechanical strength of this oxame hydrogel had increased up to ~1900%. This hydrogel system showed good swelling properties as well as rapid and independent self-healing characteristics. For tissue engineering applications, hydrogel ink with suitable yield strength, shear thinning and self-healing properties are anticipated. Chitosan methacrylate (CHMA) and polyvinyl alcohol (PVA) based pre-cross-linked hydrogel microparticles (pcHμPs) with self-healing property was developed for tissue engineering applications [217]. This hydrogel system showed outstanding shear thinning and while printing and subsequently showed self-healing characteristic after the removal of shear force. Self-supportive yield strength of pcHμPs was 540 Pa. Furthermore, this 3D printed scaffold construct showed support for the growth of stem cells and formation of cell spheroids. Various applications of self-healing hydrogels for biomedical applications are summarized in Appendix A.

## 5. Conclusions and Future Perspectives

In literature, the stability of self-healing hydrogels in physiological conditions are not explored in depth. Although, there are few reports that the self-healing hydrogels tend to lose their properties over time [7,218,219], further research is required to understand the stability of these hydrogels in physiological conditions. Furthermore, the effect of in vivo conditions, especially that of enzymes on the stability of these bonds needs to be studied in detail. Secondly, in terms of application, multifunctional self-healing hydrogels will be an interesting area of future research. One example of multifunctional system is to have both diagnostic and therapeutic factors in a self-healing hydrogel system. This could be from a combination of different nanoparticulate systems imparting multi-functionality to the system. Additionally, releasing multiple drugs/agents each targeting different pathways is another approach. Further multiple release kinetics for different agents could be incorporated into the system. For this, a complete understanding of how different dynamic bonds work on varying conditions is necessary. The combination system of drug and cells or exosomes delivery using self-healing hydrogels might get flourished in the future.

A key challenge in regenerative medicine is the requirement of the large quantity of cells for clinical use. The use of stem cells has aided in improving the efficacy of self-healing hydrogels. While it is established that stem cells encapsulated within hydrogel can maintain their stemness and enter a state of quiescence, typical quantities of cells used is low. Additionally, there are few studies that incorporate in vivo experiments of hydrogels encapsulated with stem cells. Therefore, future research into enhanced methods to encapsulate cells within hydrogels and models that better recapitulate human biological moieties is the need of the hour. To take advantage of stem cells ability to quiescence and revive them at a later stage will be beneficial to the field. A critical challenge to achieve this goal would be the supply of nutrients and oxygen to quiescent cells and designing adaptable hydrogels that promote stem cell maturation towards tissue regeneration.

Progress of self-healing hydrogel-based cartilage and bone tissue regeneration influence massive interest for future diagnostics and therapeutic strategies of bone-related defects and diseases. This progress will be regardless of the substantial prevailing challenges, including poor mechanical firmness, low integration rate, fast degradation, and immunogenicity. With an expanding knowledge and understanding of interactions between self-healing hydrogels and bone defects, this class of materials will certainly become an effective tool for cartilage and bone tissue regeneration treatment in the future.

Although hydrogels exhibit poor mechanical strength and low resistance to wear and tear, taking advantage of its promising properties like biocompatibility, flexibility and water absorbing capacity, it is predominantly used for various biomedical applications as discussed in this review. Smart self-healing hydrogels were used to overcome the aforementioned flaws, therefore improving the material’s durability and stability. As a result, it has aided in reducing discomfort in patient suffering. This review emphasizes the applications with a deep understanding of various mechanisms undergone by the biomaterials to develop into a self-healing hydrogel. The role of self-healing hydrogels in biomedical applications are a burgeoning research zone and the increasing research in this field will help to achieve plausible applications in the near future.

## Data Availability

The data presented in this study are available on request from the corresponding author.

## Appendix A

**Table A1:** Summary of self-healing mechanism and applications of various self-healing hydrogels.

S No	Hydrogel Components Involved in Bond Formation	Self-Healing Mechanism	Potential Application	Ref.
1	N-carboxyethyl chitosan and PF127	Imine bond	Wound healing	[5]
2	Guar gum, a water-soluble galactomannan with acidic poly(3,4-ethylenedioxythiophene): poly(styrenesulfonate)	Hydrogen bond	Wound healing	[6]
3	Cytosine, guanosine, modified hyaluronic acid	Hydrogen bond	Injectable drug delivery systems, tissue engineering, cell scaffold materials, and regenerative medicine	[14]
4	Polyvinyl alcohol, 3,4-dihydroxyphenyl-*L*-alanine (DOPA) and Fe complex	Hydrogen bond	Biomedical applications	[15]
5	Poly (ethylene oxide), poly(*N*-isopropylacrylamide) and ureido pyrimidinone	Hydrogen bond	3D cellencapsulation and protein delivery	[18]
6	Poly acrylic acid grafted vinyl hybrid silica nanoparticles	Hydrogen bonding and ferric ion-mediated ionic interactions	Tissue engineering	[18]
7	Cholic acid with β-cyclodextrin	Host Guest Interaction	Suitable for bio applications	[29]
8	Cationic β-cyclodextrin oligomer crosslinked by epichlorohydrin and modified by allyl glycidyl ether and glycidyl trimethylammonium chloride	Electrostatic interaction, host-guest complexation, and C-C bonds as the macrocrosslinker	Suitable for biomedical application in future	[32]
9	Cyclodextrin modified alginate, and a methacrylated gelatin	Host Guest Interaction	Suitable for biomedical application in future	[33]
10	Acryloyl-6-aminocaproic acid	Hydrophobic bond	Tissue Engineering, Biomedical application	[40]
11	Dibenzaldehyde-modified PFG_2000_	Imine bond	Biology, medicine, and engineering	[42]
12	Chitosan and dialdehyde bacterial cellulose	Imine bond	Wound healing	[45]
13	Chondroitin sulfate multiple aldehyde and N-succinyl-chitosan	Imine bond	Cell encapsulation	[46]
14	Agarose-ethylenediamine conjugate with dialdehyde-functionalized PEG	Imine bond	Tissue adhesive, Wound dressing	[48]
15	*N*,*O*-carboxymethyl chitosan/oxidized chondroitin sulfate	Imine bond	Wound healing	[50]
16	Carboxymethyl chitosan was incorporated with zinc	Imine bond	Biomedical field	[51]
17	Konjac glucomannan-chitosan	Imine bond	Wound healing	[52]
18	Quaternized chitosan and benzaldehyde-terminated poly(ethylene oxide)-b-poly(propyleneoxide)-b-poly(ethyleneoxide)	Imine bond	Drug delivery	[54]
19	Glycol chitosan and telechelic difunctional poly (ethylene glycol)	Imine bond	Wound healing	[55]
20	Aldehyde modified methyl cellulose and chitosan grafted polyethylene glycol loaded with exosome	dynamic covalent Schiff base linkage	Wound healing	[58]
21	Carboxymethyl cellulose dialdehyde and carboxymethyl chitosan	Schiff base linkage	Study the effects of paracrine signals due to cell secretion factors	[59]
22	Ablated porous PLGA grafted with hydrophobic PCL	Schiff base reaction	Bone tissue engineering application, efficient permeability and diffusion of oxygens and removal of waste from cell metabolism	[62]
23	Gelatin and oxidized dextran	Imine cross-linkage	Protection of cells from shear forces during extrusion, ECM degradation, cellular migration	[64]
24	Oxidized dextran and acid dihydrazide	Acylhydrazone bond	Wound healing	[67]
25	Polyethylene oxide and tris[(4-formylphenoxy) methyl ethane	Acylhydrazone bond	Wound Healing	[67]
26	Sodium alginate dialdehyde reacts with hydrazide	Schiff base reaction and acylhydrazone bonds	Tissue Engineering, drug delivery	[73]
27	Protein-polymer conjugates	Oxime bond	Biomedical Applications	[74]
28	Poly(vinyl alcohol) -borax gel as a matrix with dopamine-grafted oxidized carboxymethylcellulose and cellulose nanofibers	dynamic reversible borate ester linkages and hydrogen bonds along with dynamic cross-linking imine linkages	Wound healing	[114]
29	Carboxymethyl chitosan and aldehyde functionalized sodium alginate	Imine bond	Wound healing	[115]
30	Cationically charged chitosan with anionically modified bacterial cellulose	Hydrogen bond	Wound healing	[111]
31	Chitosan hydrogel cross-linked with telechelic difunctional poly (ethylene glycol) (DF-PEG-DF).	Schiff base linkage	co-delivery system for synergistic chemo-hyperthermic therapy for triple-negative breast cancer	[128]
32	Carboxyethyl-modified chitosan (CEC) and aldehyde modified hyaluronic acid (A-HA)	Dynamic Schiff base bonds between amine groups on CEC and aldehyde groups on A-HA	pH based Doxorubicin (Dox) release for cancer therapy	[129]
33	Chitosan-catechol groups	Catechol-Fe (III) coordinative interactions	Dox and Docetaxel (DTX) release for cancer therapy	[130]
34	F127–CHO (FC) [Pluoronic F127 with aldehyde group], PPR [red fluorescence-emissive polycitrate-polymine-rhodamine B polymer] and CMC [Carboxymethyl chitosan]	Schiff’s base reaction	pH-responsive Dox release for cancer therapy	[131]
35	Boric acids and diols derivatives of guanosine and isoguanosine	Boronate ester bonds	Guanosine delivery for cancer therapy	[133]
36	Hyaluronic acid was modified to have phenylboronic acid and dopamine moieties	Boronate ester bonds	Erlotinib (ERT) delivery for cancer therapy	[134]
37	Ureidopyrimidinone (UPy) side chains present in gelatin and Fe^3+^ ions	Co-ordination bond	5-fluorouracil (5-FU) delivery for cancer therapy	[135]
38	Gum Arabic (GA) was modified with multi-aldehyde group (GAMA) to be reacted with the succinic anhydride-modified CS (SCS)	Schiff base linkage	controlled release of nano curcumin as an anti-cancer treatment	[136]
39	Cellulose	Ketoester-type acyl-hydrazone bond	pH-responsive Dox release for cancer therapy	[137]
40	Amino groups in polyetherimide (PEI) and the acetoacetate groups of the four-armed star-shaped poly(2-(dimethylamino) ethyl methacrylate-co-2-hydroxyethyl methacry-late) modified with tert-butyl acetoacetate (t-BAA), SP(DMAEMA-co-HEMA-AA) and PDA NPs.	Dynamic covalent enamine bonds	Dox release for cancer therapy	[138]
41	8-arm PEG glyoxylic aldehyde and 8-arm PEG hydrazine	Glyoxylic hydrazone linkages	pH-responsive Dox release for cancer therapy	[139]
42	TPE-poly(*N*,*N*-dimethylacrylamide-stat-Diacetone acrylamide) [TPE-P(DMA-stat-DAA)]	Diacylhydrazide bonds	pH-responsive Dox release for cancer therapy	[140]
43	Pentablock terpolypeptide [PLys-b-(PHIS-co-PBLG)-PLys-b-(PHIS-co-PBLG)-b-PLys]	Pentablock peptides’ macromolecular architecture	pH- and enzyme-responsive Gemcitabine release for pancreatic cancer therapy	[141]
44	Four-arms star polymer, poly (ethylene gly-col)-b-poly(g-o-nitrobenzyl-*L*-glutamate)	Hydrophobic interaction	Light -responsive Dox release for cancer therapy	[142]
45	Amine groups of *N*-carboxyethyl chitosan (CEC) and benzaldehyde groups from poly (ethylene glycol) (PEGDA)	Covalent Schiff-base linkage	pH-dependent Dox release for hepatocellular carcinoma	[143]
46	4-arm PEG-benzaldehyde (4armPEGDA) and N-carboxyethyl chitosan (CEC)	Covalent Schiff-base linkage	pH-dependent Dox release for hepatocellular carcinoma	[144]
47	Oxidized xanthan and 8-arm PEG hydrazine	Biodegradable hydrazone linkages	Controlled release of Dox	[145]
48	α-cyclodextrin (α- CD) and poly (ethylene glycol) (PEG) chains of the poly (ethylene glycol)-block-poly (lactic acid) (PEG-b-PLA) micelles	Host-guest interaction	Controlled release of Dox against HeLa cells	[146]
49	Dialdehyde-functionalized polyethylene glycol (DF-PEG) and β-glycerophosphate (GP) cross-linked chitosan (CS) hydrogels	Schiff’s base chemistry	Controlled release of Dox in Heps tumor-bearing mice	[147]
50	Pectin aldehyde (pectin-CHO) and acylhydrazide func-tionalized polymer poly(*N*-isopropylacrylamide-stat-acylhydrazide) P(NIPAM-stat-AH)	Acylhydrazone bonds	Controlled release of two drugs: Dox and combretastatin A4 disodium phosphate (CA4)	[148]
51	Chitosan and the oxidized pectin	Imine bond formed through Schiff’s base reaction	Sustained release of 5-FU. Hydrogel with magnetic, pH- and thermo-responsive properties	[149]
52	Chondroitin sulfate multialdehyde (CSMA), branched polyethylenimine (BPEI) and BPEI conjugated graphene oxide (BPEI-GO)	Schiff-base linkage	Controlled Dox release with photothermal therapy against breast cancer	[150]
53	Benzaldehyde-functionalized pullulan (PULL-CHO) and chitosan-g-PEG (CS-g-PEG)	Schiff base linkage	synergistic magnetothermal-chemo-chemodynamic therapy for cancer	[151]
54	Grafting phenylboronic acid to the side chain of the alginate and hyaluronic acid grafted with cholesterol	Boronic ester bonds	Smart pH- and reactive oxygen species (ROS)-responsive injectable hydrogel—dual drug (amikacin and naproxen) delivery	[152]
55	Chitosan and amino acid (acryloyl-phenylalanine)	Hydrogen bonds	Controlled release of levofloxacin	[153]
56	Chitosan-cuminaldehyde	Dynamic imine bond	Controlled release of levofloxacin	[154]
57	In situ free radical polymerization of guar gum-graft-acrylic acid (GG-PAA) in presence of L-Alanine as crosslinker	Hydrogen bond	Controlled release of levofloxacin	[155]
58	Phenyl boronic acid-modified hyaluronic acid (HA–PBA) and plant-derived polyphenol-tannic acid (TA)	Boronic ester dynamic covalent bond	Dual (pH- and reactive oxygen species (ROS)) responsive silver nanoparticles based antimicrobial hydrogel	[156]
59	PLGA-PEG-PLGA co-polymeric system, comprising of a derivative of isoniazid (DINH) loaded liposomes	Hydrophobic interactions	A local drug delivery system for bone tuberculosis therapy	[157]
60	Thiol group of 4-arm-PEG-SH and silver ions	Dynamic reversible coordination bond	Acute bacterial rhinosinusitis (ABRS) therapy	[158]
61	Amine functionalized PDMS based polyzwitterionic polymersomes and polyethylene glycol dialdehyde (PEG-DA)	Schiff’s base reaction	Curcumin delivery for antimicrobial applications	[159]
62	Hyaluronic acid-g-dopamine and reduced Graphene oxide (rGO)	Physical bonding between rGO@PDA and HA-DA including hydrogen bonding, and π–π stacking	Hemostatic antioxidant conductive photothermal antibacterial hydrogels	[160]
63	Guanosine-quartet Na^+^-borate	Dynamic guanosine–borate diester bond	Controlled release of Acyclovir, an antiviral agent	[161]
64	β-cyclodextrin modified hyaluronic acid (HA-CD) and adamantane modified 4-arm-PEG (4-arm-PEG-Ad)	Host-guest interaction	Controlled release of Dexamethasone	[162]
65	Octa-cyclodextrin polyhedral oligomeric silsesquioxane (OCDPOSS) and acryla-mide-modified adamantane (Ad-AAm)	Host-guest interactions	Controlled release of Dexamethasone	[163]
66	DNA-based hydrogel crosslinked with oxidized alginate (OA) + Silica nanoparticles	Dynamic imine linkages	Sustained release of simvastatin	[164]
67	Carboxyethyl chitosan and oxidized pullulan	Dynamic imine bond	Controlled release of Dexamethasone	[165]
68	Hydroxy-propyl-methyl cellulose derivatives (HPMC-x) and PEG-b-PLA nanoparticles	Hydrophobic interactions	Dual drug delivery applications	[166]
69	Ureido-pyrimidinone (UPy) and dextran (DEX)	Hydrogen bond	Regeneration of cartilage–bone tissue complex	[199]
70	hydrazide-modified poly(L-glutamic acid) (PLGA–ADH) & alginate(catechol- and aldehyde-modified alginate, ALG–CHO–Catechol)	Hydrogen bond	Tissue engineering, bone bleeding treatment.	[205]
71	Catechol-conjugated chitosan (CHI-C)	Imine bond	Hemostasis and bone regeneration, bio-adhesive	[206]
72	chondroitin sulfate (ChS)	Acylhydrazone bond	Bone tissue engineering	[207]
73	Gelatin hydrogels with cell-infiltration and injectable (Ci-I)	Host Guest Interaction	Continuous delivery of small drugs	[209]
74	Coralline hydroxyapatite (CHA)/silk fibroin (SF)/glycol chitosan (GCS)/difunctionalized polyethylene glycol (DF-PEG)	Imine bond	Bone defect treatment	[210]
75	PEG hydrogel incorporated with adhesive liposomes (A-LIP) loaded with BMP-2	Disulfide bond	Osteoporotic fractures and bone marrow cavity treatment	[211]
76	polysaccharide matrices & LAPONITE^®^ (LAP) nanoplatelets	Hydrogen bond	Orthopedic treatment	[212]
77	Mg^2+^/curcumin, QCS and Pluronic^®^F127 (PF127-CHO)	Imine bond	Tendon-to-bone healing	[213]
78	PVA based hydrogel	Hydrogen bond	Multiple applications	[215]
79	poly(*N*-hydroxyethyl acrylamide-co-methyl vinyl ketone) (PHEAA-co-PMVK) with a bifunctional hydroxylamine.	Hydrogen bond	Tunable mechanical property for various applications	[216]
80	Chitosan methacrylate (CHMA) and polyvinyl alcohol (PVA)	Hydrogen bond	Bone tissue engineering	[217]
81	Fluorenylmethoxycarbonyl-diphenylalanine (Fmoc-FF), Fmoc-RGD (arginine-glycine-aspartate)	Hydrogen bonds	Promotion of cell adhesion	[220]
